# Deep Blue and Highly Emissive ZnS-Passivated InP QDs: Facile Synthesis, Characterization, and Deciphering of Their Ultrafast-to-Slow Photodynamics

**DOI:** 10.1021/acsami.2c16289

**Published:** 2023-01-06

**Authors:** Soumyadipta Rakshit, Boiko Cohen, Mario Gutiérrez, Ala’a O. El-Ballouli, Abderrazzak Douhal

**Affiliations:** Departamento de Química Física, Facultad de Ciencias Ambientales y Bioquímica and INAMOL, Universidad de Castilla-La Mancha, Avenida Carlos III, Toledo 45071, Spain

**Keywords:** InP QDs, surface passivation, emission enhancement, charge carriers, ultrafast dynamics, deep trap states

## Abstract

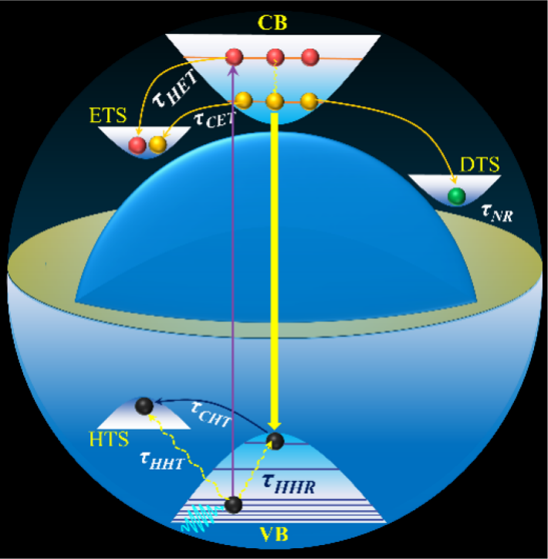

InP-based quantum dots (QDs) are an environment-friendly alternative to their heavy metal-ion-based counterparts. Herein we report a simple procedure for synthesizing blue emissive InP QDs using oleic acid and oleylamine as surface ligands, yielding ultrasmall QDs with average sizes of 1.74 and 1.81 nm, respectively. Consecutive thin coating with ZnS increased the size of these QDs to 4.11 and 4.15 nm, respectively, alongside a significant enhancement of their emission intensities centered at ∼410 nm and ∼430 nm, respectively. Pure phase synthesis of these deep-blue emissive QDs is confirmed by powder X-ray diffraction (PXRD), X-ray photoelectron spectroscopy (XPS), and transmission electron microscopy (TEM). Armed with femtosecond to millisecond time-resolved spectroscopic techniques, we decipher the energy pathways, reflecting the effect of successive ZnS passivation on the charge carrier (electrons and holes) dynamics in the deep-blue emissive InP, InP/ZnS, and InP/ZnS/ZnS QDs. Successive coating of the InP QDs increases the intraband relaxation times from 200 to 700 fs and the lifetime of the hot electrons from 2 to 8 ps. The lifetime of the cold holes also increase from 1 to 4 ps, and remarkably, the Auger recombination escalates from 15 to 165 ps. The coating also drastically decreases the quenching by the molecular oxygen of the trapped charge carriers at the surfaces of the QDs. Our results provide clues to push further the emission of InP QDs into more energetically spectral regions and to increase the fluorescence quantum yield, targeting the construction of efficient UV-emissive light-emitting devices (LEDs).

## Introduction

Quantum confined semiconductor nanostructures having diameters within 10 nm, also known as quantum dots (QDs), are among the most compelling examples of how materials can demonstrate exciting behavior at the nanoscale.^1,2^ The quantized and discrete energy states of the free charge carriers within these materials enable them to show tunable physical, electrical, and optical properties.^2,3^ The bright narrowband and tunable emission across the UV–vis–NIR region, alongside their excellent photostability, made QDs one of the most promising materials for photovoltaic and optoelectronic applications.^3−9^ Although CdSe, CdS, CdTe, and PbS are the most studied and popular QDs, their severe toxicity is a significant drawback impeding their commercialization and regulation.^6,10^ Indeed, for practical and sustainable commercialization of QDs in display and energy harvesting industries, the development of Cd-free QDs is a matter of urgent necessity. Among heavy metal free QDs, indium (In)-based ones have attracted much attention lately for their high quantum yield, emission tunability, and low toxicity, becoming an outstanding alternative in optoelectronic and imaging applications.^11^ Although red and green indium phosphide (InP)-based QD light-emitting devices (QLEDs) show considerable external quantum efficiencies (EQE) of 20%^12^ and 7%,^13^ respectively, the progress on blue emissive InP QDs is far behind with an EQE of ∼2.3%.^14,15^ Blue LEDs are an utmost necessity for the display industry as they can be doped (or coated) with other phosphors for achieving efficient full-color lights.^4^ Engineering the core/shell interface to get spontaneous amplified blue emission remains a challenge due to its high threshold value. For blue emissive core/shell QDs, the potential barrier between the core and the shell is very small, which renders easy charge carrier tunneling into surface defects, consecutively decreasing the photoluminescent quantum yield (PLQY) and hindering photonic applications.^16^ Most of the InP QDs reported hitherto have shown an emission intensity maximum centered at 480 nm,^14,15,17,18^ with one singular exception where the emission maximum appears at 465 nm.^19^ Very recently weak emissive InP clusters with emission maxima 410 nm has been reported.^20^ Since the bandgap of bulk InP is 1.35 eV, its adjustment to the required 2.9–3.1 eV is rather challenging. This fact and the inherent low photoluminescence quantum yield (PLQY) of InP QDs originating from their intrinsic trap induced defects are the major drawbacks that require tackling for successful deployment of InP QDs in the fabrication of efficient optoelectronic devices.^21^ Surface etching and surface passivation by a larger bandgap shell material are well-established strategies that increase the PLQY of InP QDs.^11,14,19,17,21,22^ However, the recent employment of small molecules at the surface of InP QDs enhanced PLQY and extended photoluminescence (PL) lifetime significantly.^23,24^ From the point of view of fundamental science, a deep understanding of the carrier trapping in QDs alongside concurrent multiexciton generation (MEG) is paramount for designing and developing efficient optoelectronic devices. The involvement of excitonic defects and trap states renders them quite appealing for exploring the exciton dynamics that shape their photophysical behavior. Although some studies have been done on the charge carrier dynamics of red and green emissive InP QDs, to the best of our knowledge, the photodynamics of blue emissive InP QDs remain unexplored so far.^21,22,25,26^

Herein, we report the synthesis of deep-blue emissive ultrasmall InP QDs (emission intensity maxima at ∼400 and ∼419 nm) using oleic acid (OAC) and oleylamine (OAM) as surface ligands. Subsequently, we coated the InP core with a thin ZnS shell, which increased the PLQY from 5 to 37% for OAC-based QDs and from 6 to 45% for OAM-based QDs, while the PL lifetime increased from ∼1 ns to ∼3 ns for both ligands. The as-synthesized QDs have been characterized by powder X-ray diffraction, X-ray photoelectron spectroscopy, and high-resolution transmission electron microscopy to determine their purity and crystallinity. We then investigated how the ZnS shell improved the PLQY by performing a detailed photodynamics characterization using fs–ms time-resolved absorption and emission techniques. We explored the effect of surface passivation on carrier trapping, relaxation, and Auger recombination in the coated and uncoated InP QDs. We observed that adding consecutive ZnS shells delays both electron and hole relaxation and effectively increases the time constants of hot and cold electrons relaxations from 2 to 8 ps and 5.8 to 66 ps, respectively, while that of cold holes trapping increases from 1 to 4 ps. Auger recombination slowed down from 15 to 165 ps upon surface passivation. Flash photolysis experiments revealed photogenerated inactive surface traps, which were evident in a 0.20 μs nonradiative recombination time alongside deep trap states below the conventional emissive states that relax in 1.2 μs. Our findings decipher the photoevents occurring in the fs–ms regime for bare and ZnS-coated InP QDs, offering a cumulative picture of the different relaxation pathways and demonstrating how the surface passivation by the ZnS shell largely enhances the PLQY of the bluest emissive InP QDs, hitherto reported, thus paving the way for engineering more efficient deep-blue LEDs.

## Results and Discussion

### Synthesis and Structural Analysis

**Figure 1:**
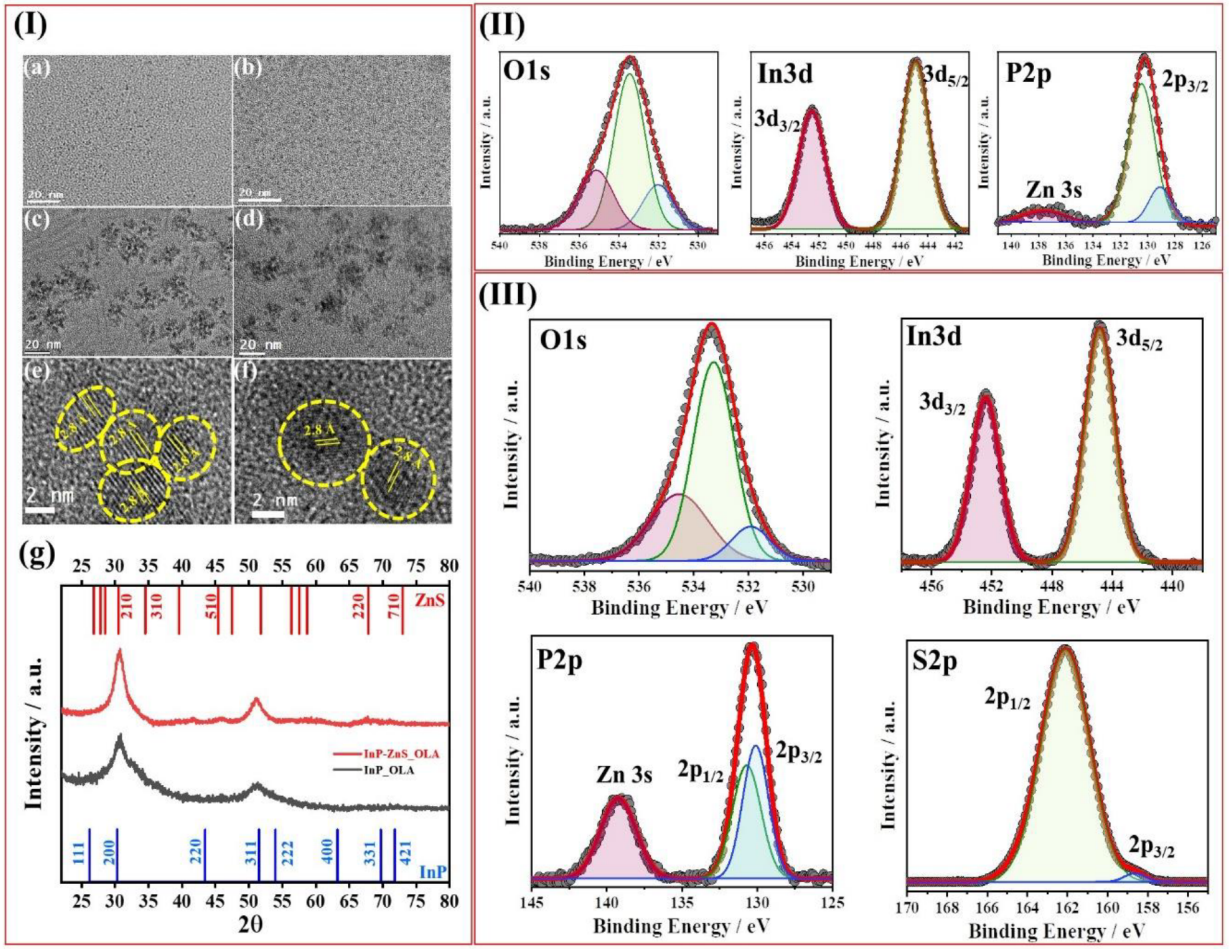
(I) Wide scale TEM images of InP core (a, b) and InP/ZnS/ZnS QDs (c, d) with surface ligands OAC (a, c) and OAM (b, d), respectively. (e) and (f) represent HRTEM images of the InP/ZnS/ZnS QDs with surface ligands OAC and OAM respectively. (g) X-ray diffraction patterns of the InP core and InP/ZnS/ZnS QDs with surface ligand OAC. (II) High resolution XPS scans of O 1s, In 3d, Zn 3s, P 2p, S 2p for InP core QDs. (III) High resolution XPS scans of O 1s, In 3d, Zn 2p, S 2p and for InP/ZnS/ZnS QDs with surface ligand OAC. For any element red represents the highest binding energy, whereas lower and lowest ones have been represented in green and blue.

In the first step of QD characterization, morphological analysis was carried out using high-resolution transmission electron microscopy (HRTEM). Figure 1I shows the HRTEM images of bare and double ZnS-coated InP QDs with surface ligand OAC and OAM. The images show InP QDs with a size of 1.74 ± 0.21 nm and 1.81 ± 0.25 nm (Figure 1Ia,b and Figure S1) in the presence of OAC and OAM, respectively. With consecutive coating, the QDs size increased to 4.11 ± 0.6 nm for OAC and 4.15 ± 0.8 nm for OAM, respectively (Figure 1Ic,d). The Gaussian size distribution fittings clearly showed that the mean QD diameter increased upon consecutive ZnS coating (Figure S1I). The HRTEM images of the double coated QDs revealed well-defined crystal plane striations, indicating an excellent degree of crystallinity. An interplanar distance of 2.8–2.9 Å was measured for InP/ZnS/ZnS QDs, which can be readily indexed to (200) lattice planes that correspond to the zinc blend crystal structure (Figure 1Ie,f). However, no evidence was observed regarding an interface between the core and the shell, which implies that the shell growth process probably occurred in the coherent epitaxial region and excludes the possibility of independent homogeneous nucleation of the shell precursors.^27^

The InP QDs with surface ligand OAC were further characterized by powder X-ray diffraction (PXRD). The reflection peaks of the ultrasmall InP core QDs were broad due to their extremely small size (Figure 1Ig), which is consistent with the TEM observation.^17,28^ The strong diffraction peaks located at 2θ of 30.51 and 50.1 correspond to (200) and (311) planes depicting a zinc blend phase.^15,20,29^ The diffraction peaks were sharper for the InP/ZnS/ZnS QDs compared to InP QDs, which also indicates effective size inflation by epitaxial shell overgrowth.^28,15^ Minimal contributions around 40°, 46°, and 68° and between 55° and 60° arose, which could be ascribed to ZnS coating.^30^ Due to the presence of Zn^2+^ in the core and ZnS as the coating, the overall diffraction peak positions also shifted to slightly higher 2θ values.^28^

Next, X-ray photoelectron spectroscopy (XPS) was employed to characterize the chemical states of the core and coated InP QDs with surface ligand OAC. The XPS spectra and relative data of the as-synthesized QDs are shown in Figure 1II and Figure S1II, respectively, while the XPS spectra and relative data of InP/ZnS/ZnS QDs are depicted in Figure 1III and Figure S1III, respectively. As evident from both survey spectra for InP and InP/ZnS/ZnS QDs, the atomic percentage of C is very high due to the presence of oleic acid at the surface of these QDs. The C 1s core peaks for the ultrasmall InP cores present three components at 284.90 eV, 286.2 eV, and 288.2 eV (Figure S1II). The first strongest component can be ascribed to the sp^3^ carbons regarding C–C and C–H bonds due to the presence of oleic acid at the surface. The second component corresponds to the C–O bonds, whereas the third component is associated with carboxylate functional group. Minimal shifts in the case of coated InP QDs may be ascribed to the change in the chemical environment.^29^ The high-resolution XPS spectra further demonstrated the In 3d_5/2_ and In 3d_3/2_ peaks at 444.6 and 452.2 eV (Figure 1II), which are almost identical to the typical values for bulk crystalline InP which further confirms the presence of pure phase InP cores.^29,15,30^ For the coated QDs, the In 3d_5/2_ and In 3d_3/2_ peaks appeared exactly at the same positions as in InP cores, i.e., at 444.6 and 452.2 eV (Figure 1III). In addition, the sharp In 3d_5/2_ and In 3d_3/2_ peaks of the coated and uncoated InP QDs suggest that the peaks are attributed to a single chemical bonding state of the In–P bond.^30^ The P 2p spectrum of the InP cores with OAC surface ligand gives a strong peak centered at 130.2 eV and a very weak peak at 138.1 eV (Figure 1II). The high-intensity peak of the binding energy in the range 128–131 eV is related to the P^3–^ for the bare InP. The strongest peak can be deconvoluted with a doublet at 129.1 and 130.9 eV corresponding to P 2p_3/2_ and P 2p_1/2_. Although we did not any see any separate peak for oxidation, the surface oxidation signature is within the broad nature of the peak. For the coated QDs, a sharp and strong peak arose at 130.2 eV, which can also be deconvoluted with a doublet at 129.8 and 130.4 eV corresponding to P 2p_3/2_ and P 2p_1/2_. The values of the spin–orbit splitting between P 2p_3/2_ and P 2p_1/2_ in the case of bare InP QDs and ZnS coated InP QDs were 1.8 and 0.6 eV. This reduced spin orbit splitting is a direct evidence of the reduction of surface oxidation of the InP QDs.^15,29,31^ For the InP cores a very small Zn 3s peak at ∼139 eV was observed due to the initial addition of 5% Zn^2+^ during the synthesis of the cores. However, compared with InP cores, InP/ZnS/ZnS QDs revealed a much stronger peak at 139.2. (Figures 1III and S1III).^30,31^ The S 2p spectrum predominantly gives a single contribution centered at 162.1 eV which is associated with S^2–^ present in ZnS. Although we have confirmed the presence of pure phase ultrasmall InP core QDs, the strong oxygen peak at 533.3 eV arose from the carboxylate group of the ligand as supported by the C 1s peaks. Apart from analyzing the In 3d, O 1s, P 2p, and C 1s core peaks, the Auger peak M_4_N_45_N_45_ was also investigated and the signature in both InP and InP/ZnS/ZnS QDs was the same, which confirms the absence of any In_2_S_3_ formation.^29^

### Steady-State Measurements

The as-synthesized InP QDs with OAC and OAM as surface ligands showed absorption maxima at 344 and 345 nm, respectively (Figure S2Ia,b). To rationalize the sizes of the InP cores with reference to their emission maxima, we compared our TEM results with previously reported InP cores having different emission wavelength maxima (see Supporting Information Table S1, Figure S3).

**Figure 2:**
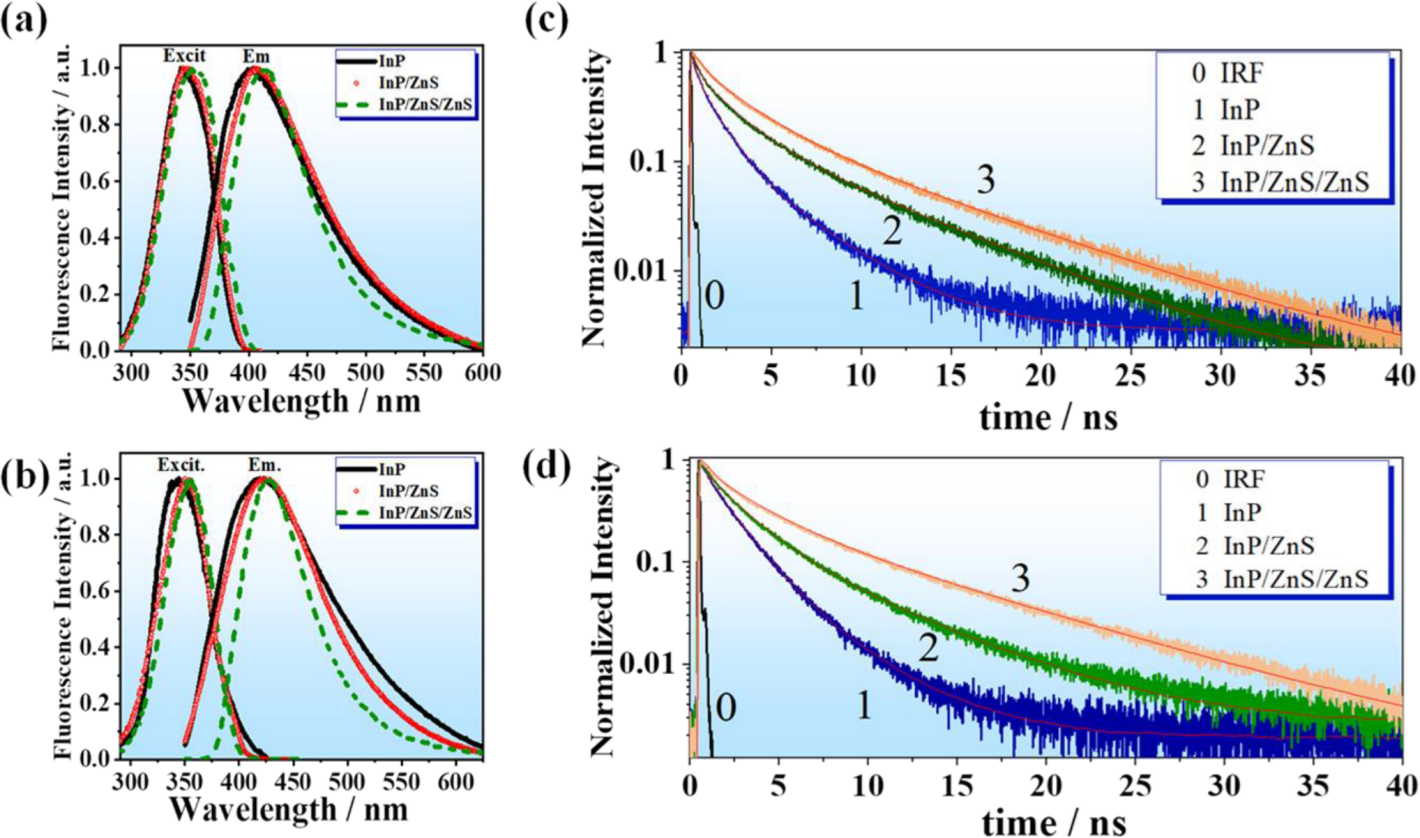
Excitation and emission spectra of InP (black line), InP/ZnS (red dotted line), and InP/ZnS/ZnS (green dashed line) QDs containing (a) OAC and (b) OAM as surface ligands. The excitation wavelength for InP and InP/ZnS QDs is 330 nm, whereas for InP/ZnS/ZnS QDs it was 340 nm. For the excitation spectra, the observation wavelengths were (a) 425 nm and (b) 450 nm, respectively. Magic-angle time-resolved PL decays of InP, InP/ZnS, and InP/ZnS/ZnS QDs with (c) OAC and (d) OAM as surface ligands, monitored at their emission intensity maxima, upon excitation at 371 nm. The instrument response function (IRF) is 70 ps. The solid lines are from the best global fit using a multiexponential function.

After the subsequent ZnS coating, the absorption maximum of the InP/ZnS QDs was shifted to 351 nm for OAC and 355 nm for OAM. Figure 2a,b depicts the excitation and emission spectra of InP, InP/ZnS, InP/ZnS/ZnS QDs in hexane using OAC and OAM. The unpassivated InP QDs exhibit a deep blue emission with a maximum at 398 and 419 nm for OAC and OAM, respectively. To rule out the presence of any In- or P-based inorganic chemical impurity, three different solutions were prepared and examined for each surface ligand. For surface ligand OAC, the three solutions consisted of (i) OAC and ODE, (ii) OAC, In(OAc)_3_, and ODE, and (iii) OAC, TDMAP, and ODE. The solutions were heated at 170 °C for 2 h, and then the resulting mixtures were isolated by ethanol and redispersed in hexane for further steady-state fluorescence measurements. The same procedure was implemented in the case of the other surface ligand OAM where the three solutions were (i) OAM and ODE, (ii) OAM, In(OAc)_3_, and ODE, and (iii) OAM, TDMAP, and ODE. The recorded spectra are illustrated in Figure S2II, and these confirm that the emission spectra shown in Figure 2 do not originate from any inorganic impurities that might be produced during the synthesis. Uncoated InP QDs with surface ligand OAC and OAM show broad emission centered at 398 and 419 nm. The broad emission of uncoated InP QDs is well documented and can be attributed to the hole trap-assisted emission and strong electron–phonon coupling.^21,22^ Recently, consecutive ZnS coating resulted in a small bathochromic shift of the excitation and emission maxima (Figure 2). For OAC with two consecutive ZnS shells, the emission intensity maxima shifted from 398 to 410 nm, whereas for OAM the shift was from 419 to 430 nm (Figure 2 a,b). Similarly, the excitation spectra of the corresponding QDs revealed a red-shift upon coating with ZnS shell. As shown in Figure 2a,b, the maxima of the excitation spectra shifted from 344 to 351 nm for OAC-based QDs and from 345 to 355 nm for OAM-based QDs. Consecutive ZnS shell addition also results in a decrease in the full width at half-maximum (fwhm) of the PL spectra from 90 to 65 nm for OAC and from 85 to 50 nm for OAM, which indicates improved color purity upon ZnS coating.^18^ The decrease in the fwhm values for InP QDs having ZnS shell coating agrees with previous reports on CdSe and InP QDs and can be ascribed to a narrower particle size distribution and stronger exciton localization.^17,22,27,32^ However, we cannot exclude the possibility of carrier delocalization from the core into the ZnS shell due to the small energy bandgap difference between the core and shell materials. Therefore, the small red-shifted emission of the bare InP QDs can be attributed to the possible spreading of the electron wave function through the small energy barrier between the core and shell material and the formation of a slight quasi type-II structure (Scheme S2).^33−37^ However, it is evident that the effect of carrier confinement is prevalent rather than the leakage of the excitons into the shell.

The PLQY of the uncoated InP QDs with OAC ligand was 5%, whereas for those having OAM ligand it was 7%. Remarkably, the PLQY values increased to 18 and 21% for the single-coated QDs and to 37 and 45% for the double-covered InP/ZnS/ZnS QDs. These increments in the PLQY values of InP QDs with consecutive ZnS coating indicate a rapid increase in the radiative transition and a decrease in the nonradiative transitions within the InP QDs.^17^ The average radiative (⟨*k*_r_⟩) and nonradiative (⟨*k*_nr_⟩) rate constants were calculated by the equations depicted in SN1 (Supporting Information). Table 1 summarizes the obtained values that clearly show that passivating the QD surface with ZnS made nonradiative channels less efficient and improved radiative recombination rates. Similarly, increments of the radiative rate constants have been previously reported for InAs and CdSe QDs upon passivating their surface.^38,39^ Comparatively, surface etching of InAs QDs with HF increased the values of the radiative constants, and therefore the PLQY, and decreased the oscillator strength, which has also been substantiated by longer lifetimes.^38^ The increase in the radiative rate constant with the addition of consecutive ZnS shells can thus be attributed to the stronger confinement of the carriers in the core material.^38−40^

**Table 1:** Values of the Absorption and Emission Intensity Maxima, Average PLQY, Average PL Lifetimes along with Radiative and Nonradiative Rates of the Uncoated and Coated InP QDs^a^

ligand	QD	λ_max_^abs^ (nm)	λ_max_^em^ (nm)	Φ	⟨τ_avg_⟩ (ns)	⟨*k*_r_⟩ (10^7^ s^–1^)	⟨*k*_nr_⟩ (10^8^ s^–1^)
OAC	InP	344	398	0.05	0.93	5.38	10.21
	InP/ZnS	349	405	0.18	1.95	9.23	4.22
	InP/ZnS/ZnS	351	410	0.37	2.47	14.98	2.55
OAM	InP	345	419	0.07	1.08	6.48	8.61
	InP/ZnS	351	425	0.21	2.16	9.72	3.66
	InP/ZnS/ZnS	355	430	0.45	3.05	14.75	1.80

a The estimated errors are around 10–15%.

### Picosecond Time Resolved Measurements

To get better insight into the emission enhancement of InP QDs due to ZnS coating, we recorded the emission decays at different observation wavelengths upon excitation at 371 nm (Figure 2c,d and Figure S4). The decays were fitted using a multiexponential global fit with three time components. Table 2 illustrates the values of the lifetimes (τ_*i*_), normalized (to 1) pre-exponential factors (*a_i_*), and contributions (*c_i_*) of each component in the decays. The lifetimes for the InP QDs with surface ligand OAC were τ_1_ = 0.17 ns, τ_2_ = 1.31 ns, and τ_3_ = 4.08 ns (Table 2), while for the OAM-based QDs the corresponding time components were τ_1_ = 0.21, τ_2_ = 1.52, and τ_3_ = 4.92 ns. The addition of the first ZnS shell increased the values of τ_1_, τ_2_, and τ_3_ to 0.24, 1.97, and 5.71 ns and to 0.30, 2.05, and 6.63 ns for the OAC and OAM QDs, respectively. Upon the second ZnS passivation, the values of τ_1_, τ_2_, and τ_3_ further increased to 0.43, 2.46, and 7.68 ns and to 0.60, 2.95, and 8.71 ns, respectively. The slight difference in the values of the time components for the single and double-ZnS shell QDs using the two surface ligands can be explained in terms of difference in the number of intrinsic defects of the core InPs QDs. Thus, even if the passivation by the ZnS shell/s is comparable, the number of intrinsic defects in the core QDs will remain different giving rise to the observed behavior.^21,26^ To better characterize the multiple recombination pathways, the dependence on gated wavelength of the PL decay was monitored. When the PL decays of the samples were recorded at lower energies, the relative contribution of τ_1_ remained almost constant, whereas that of τ_2_ slightly decreased, and the contribution of τ_3_ increased (Table S2 and Table S3). Although the origin of the multiexponential decays for the InP QDs is not yet fully understood, it indicates the involvement of several relaxation pathways. But it is very clear from the decay curves that growth of ZnS shell leads to an overall suppression of the nonradiative fast decay components. Therefore, we focused instead on the average lifetime (τ_avg_) of the individual systems, and with both ligands the QDs experienced ∼3 times increase upon surface passivation with ZnS. This phenomenon also indicates that the ZnS shell can passivate the related surface traps.^41−43^ However, we also expect the Auger recombination to play a pivotal role. Trap-assisted Auger process is considered one of the culprits for low emission in bare QDs.^44,45^ Surface passivation with ZnS decreases the trap states, leading to reduced trap-assisted nonradiative Auger recombination and hence emission enhancement.^26,46^ The effect of the Auger recombination is discussed later in more detail. Thus, the increment of all the time components upon ZnS coating can be interpreted in terms of passivation of the surface defects to result in a better exciton localization in the core, which leads to longer radiative transition times.^42,47,48^

**Table 2:** Values of Time Constants (τ_i_), Normalized (to 1) Pre-Exponential Factors (*a*_*i*_), and Contributions (*c_i_* = τ_*i*_ × *a_i_*/∑(τ_*i*_ × *a_i_*)) in the Signal Obtained from a Global Multiexponential Fit of the Emission Decays of QDs upon Excitation at 371 nm^a^

ligand	QD	τ_1_ (ns)	*a*_1_	*c*_1_	τ_2_ (ns)	*a*_2_	*c*_2_	τ_3_ (ns)	*a*_3_	*c*_3_
OAC	InP	0.17	0.53	0.10	1.31	0.39	0.56	4.08	0.08	0.35
	InP/ZnS	0.24	0.36	0.05	1.97	0.48	0.47	5.71	0.16	0.48
	InP/ZnS/ZnS	0.43	0.38	0.06	2.46	0.47	0.47	7.68	0.15	0.47
OAM	InP	0.21	0.51	0.12	1.52	0.38	0.54	4.92	0.08	0.34
	InP/ZnS	0.30	0.38	0.07	2.05	0.48	0.47	6.63	0.16	0.46
	InP/ZnS/ZnS	0.60	0.30	0.05	2.95	0.47	0.44	8.71	0.17	0.51

a The observation emission wavelength was 425 nm. The data are within 10–15% error limit.

### Ultrafast Fluorescence Measurements

**Figure 3:**
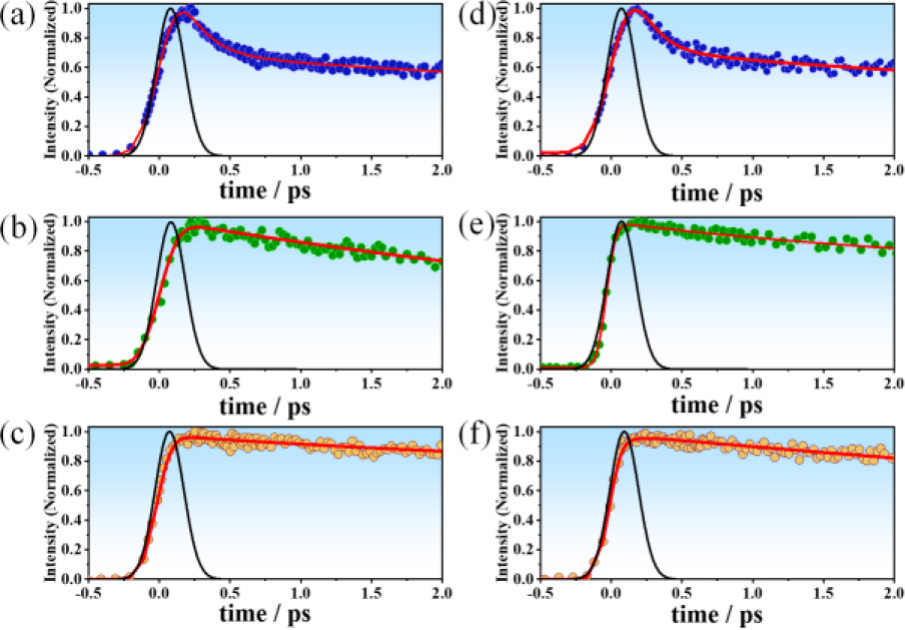
Femtosecond emission transients of InP, InP/ZnS, and InP/ZnS/ZnS QDs with OAC (a–c) and OAM (d–f) as surface ligands upon excitation at 360 nm. The observation wavelength was 425 nm. The red solid lines are from the best fits using a multiexponential function, and the black solid line is the IRF (∼180 fs).

To explore the efficacy of the surface passivation by the ZnS shell over the InP QDs with the two surface ligands, we studied the charge carrier dynamics by femtosecond fluorescence up-conversion. Figure 3 shows the fluorescence up-conversion transients gated at 425 nm that correspond to the band-edge emission of the coated and uncoated InP QDs. All the emission signals were fitted by a biexponential function plus a constant offset (Table 3). To begin with, the decays of both uncoated InP QDs with surface ligands OAC and OAM show an ultrafast component with a value of 0.5 and 0.6 ps, respectively (Figure 3a,d and Table 3), followed by a longer component of 5.8 and 8.2 ps and by an offset.

**Table 3:** Values of Time Constants (τ_i_) and Normalized (to 1) Contributions (*c_i_* = τ_*i*_ × *a_i_*/∑(τ_*i*_ × *a_i_*)) (*a_i_*) Obtained from a Multiexponential Fit of the Femtosecond Emission Transients of InP, InP/ZnS, InP/ZnS/ZnS QDs with OAC and OAM as Surface Ligands upon Excitation at 360 nm^a^

ligand	QD	τ_1_ (ps)	*c*_1_	τ_2_ (ps)	*c*_2_	τ_3_ (ns)	*c*_3_
OAC	InP	0.50	0.52	5.80	0.26	>1	0.22
	InP/ZnS	1.70	0.35	32	0.35	>1	0.30
	InP/ZnS/ZnS	7.50	0.25	46	0.35	>2	0.40
OAM	InP	0.60	0.50	8.20	0.30	>1	0.20
	InP/ZnS	2.45	0.34	35	0.36	>1	0.30
	InP/ZnS/ZnS	9.30	0.23	66	0.37	>2	0.40

a The monitored emission wavelength is 425 nm. The data are within 10–15% error limit.

Since this technique has the ability to monitor the radiative recombination of the excitons, increment of PLQY via arresting the carrier trapping at the surface can readily be recognized. It has been proven that due to the higher density of states, hole energy level differences could be resonant with ligand vibrational modes to result in a very fast hole relaxation near the bandgap, whereas for electrons it is much slower.^49,50^ Several studies have reported that unpassivated InP QDs have unsaturated phosphorus dangling bonds that form a lower energy state over the VB and trap the holes rapidly.^23,43,51−54^ Therefore, we attribute the ultrafast component to the trapping of the photogenetared holes (τ_CHT_). For bare InP QDs with surface ligand OAC and OAM, the contributions of this hole trapping are around 50% which can be directly linked with their poor PLQY. With successive coating the shortest time component increased (for OAC) from 0.5 ps (bare) to 1.7 ps (first shell) and 7.5 ps (second shell). A similar trend was observed for the OAM where the values changed from 0.6 ps (bare) to 2.45 ps (first shell) and 9.3 ps (second shell). We explain this increase with successive ZnS shell addition along with the decrease in the charge carrier trapping due to the passivation of the surface trap states. On the other hand, the relative contribution of this component (*c*_1_) decreases with each consecutive addition of ZnS shell for both types of QDs, which suggests that the VB offset between the core and shell materials acts as a hole well where the confinement of the hole within the core gets stronger following the addition of consecutive shells. Therefore, the passivation of the InP QD surface through ZnS coating makes the hole trapping phenomena less prevalent.^26,55^ Although the small difference in the values of τ_CHT_ for uncoated and coated QDs using the different ligands could be explained in terms of different number of intrinsic defects in the core InP, most probably this effect is less significant in the ultrafast dynamics since the values are almost within the experimental error (10–15%). Previous reports on InP,^56^ CdSe,^57,58^ and CdS^59^ QDs have also suggested that the hole trapping process occurs on time scales of 1–5 ps based on the abundance of the hole trapping states. However, and in agreement with our observations, faster hole trapping in InP QD has been reported^51^ and was attributed to unpassivated, anion-rich, dynamic InP surface.^52,54^

Apart from the trapped holes, the PLQY of QDs also gets affected by trapping of the relaxed electrons at the band edge state. Since we are looking at the recombination from the band edge state, the lower density of states in the CB leads to longer lifetime of the relaxed electrons at the CB than the holes at VB.^50^ Thus, we assign the comparatively slower second component to cold electron trapping (τ_CET_) by the unsaturated and undercoordinated atoms on the InP QD surface that serve as efficient electron traps.^43,51,60^ The time scales for τ_CET_ are in agreement with those previously reported for InP,^56^ CdSe,^61,57^ and CdS^62^ QDs. The values of this component show a similar trend as the one observed for τ_CHT_, with a significant increase from 1.7 (OAC)–2.4 (OAM) ps to 32 (OAC)–35 (OAM) ps for the single-coated InP/ZnS QDs and to 46 (OAC)–66 (OAM) ps for the double-coated InP/ZnS/ZnS (Table 3 and Figure S5). The relative contribution of this component shows weak dependence on the presence of the ZnS shells. Since the energy bandgap difference between the core and shell material is small, quasi type-II behavior can also be observed along with type-I characteristics. For the bare InP QDs, the charge carriers are delocalized throughout the QDs, but with the addition of the ZnS coating the VB offset confines the holes, whereas the low band offset of the CB (0.2 eV) confines the electrons showing type-I behavior. However, there still exists the possibility of delocalization of the electrons into the ZnS shell giving rise to partial quasi type-II properties.^63−65^ Similar effects of surface passivation with ZnS and ZnSe on the hole and electron trapping processes for green and red emissive InP QDs have been reported.^22,43,66^ We also observed a moderate change in the value of τ_CET_ when the surface ligand was changed from OAC to OAM, which can be explained by the difference in electronegativity between OAM and OAC.^55^

The emission decays also revealed a constant offset corresponding to the radiative relaxation (τ_RR_) of the excitons as characterized and discussed in the previous section. Importantly, consecutive surface passivation caused an increase in the contribution of τ_3_ that exceeds the gain noted in the other components. Finally, we did not observe any wavelength dependence of the femtosecond emission transients both at short and long-time scales (Figures S6 and S7).

### Femtosecond Transient Absorption Measurements

**Figure 4:**
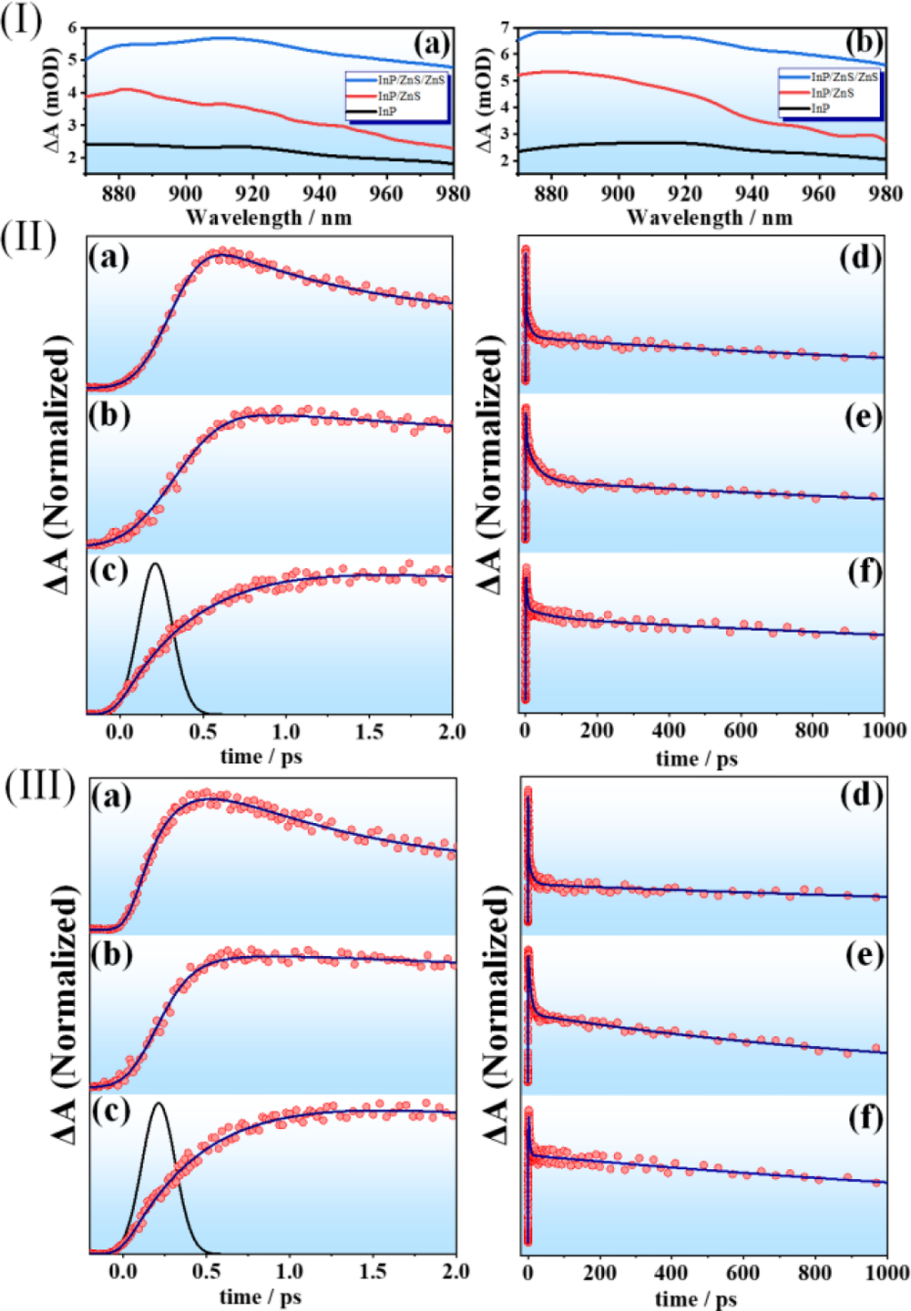
(Ia) and (Ib) represent the NIR TA spectra of InP, InP/ZnS, and InP/ZnS/ZnS QDs at 1 ps delay with surface ligand OAC and OAM, respectively. (II) Early time TA dynamics probed at 920 nm upon 340 nm excitation of (a) InP, (b) InP/ZnS, and (c) InP/ZnS/ZnS QDs using OAC as surface ligand. Full-scale data for the InP, InP/ZnS, and InP/ZnS/ZnS QDs are displayed in (d), (e), and (f), respectively. (III) Early time TA dynamics probed at 920 nm upon 340 nm excitation of (a) InP, (b) InP/ZnS, and (c) InP/ZnS/ZnS QDs with OAM as surface ligand. Full-scale data for the InP, InP/ZnS, and InP/ZnS/ZnS QDs are shown in (d), (e), and (f), respectively. The solid lines are from the best fits using a multiexponential function.

Next, we performed femtosecond transient absorption (fs-TA) measurements to decipher the nonradiative processes operating in the charge carrier dynamics of the uncoated and coated blue emissive InP QDs. Upon exciting at 340 nm (i.e., the band-edge excitation for the coated and uncoated QDs), we probed the visible (430–620 nm) and the NIR (870–1000 nm) spectral regions. Unlike the ultrafast fs-TA signal in the visible region and the PL measurements, the observed NIR signal can be directly linked to the absorption of probe photons by individual charge carriers.^67,68^ Since we could not get the bleach signal in the fs-TA measurements, we started probing the NIR region. In this region, we can monitor the 1S_e_–1S_p_ intraband transition devoid of ground state and biexcitonic features.^67,68^ Earlier studies have probed the NIR region either to get knowledge about intraband transitions^67−70^ or to monitor the electron transfer dynamics.^71−73^ However, we cannot completely exclude the contribution from photogenerated holes to the NIR signal. We observed a broad and featureless weak signal for both the uncoated and the ZnS-coated InP QDs (Figure 4I and Figure S8). Figure 4I presents the spectra of all the QDs at 1 ps delay. No probe wavelength dependence for the decays associated with the broad signal in the NIR region was noted at 870, 920, and 980 nm (Figures 4II, 4III, and S9). The multiexponential fit to the decays probed at 920 nm gives three components: a fast fs-rise component, a fast ps-decay, and a long ns-decay (Table S4).

Regardless of the ligands employed, the bare InP QDs showed an ultrafast rise of 0.2 ps, followed by a fast decay of 2 ps. The rise time for InP/ZnS and InP/ZnS/ZnS QDs increased from 0.4 to 0.7 ps for both OAC- and OAM-based QDs. Consequently, the fast decay component got slower to 5 and 8 ps for InP/ZnS and InP/ZnS/ZnS QDs, respectively (Table S4). All the decays also revealed a long component (>1 ns) that was not completely resolvable within our experimental delay window. The positive signal in the NIR region arises from the promotion of the electrons in the CB-related states due to absorption of the probe. With consecutive coating of the InP QDs, the number of trap states and the efficiency of the nonradiative decay pathways at the surface are reduced, and therefore more electrons can be promoted at higher states. The increase in the initial rise time in the kinetics can thus be related to the promotion of the accessible electrons in the CB state (τ_IBR_).^72,73^ Note that the value of this component is most probably affected also by the hot hole relaxation process. This is further substantiated by the behavior of the fast decay, which we ascribe to hot electron trapping (τ_HET_) in surface trap states. The lifetimes of the trapped hot electrons were 2, 5, and 8 ps for the bare, single-coated, and double-coated InP QDs, respectively, independent of the surface ligand. Similar lifetimes for the hot electrons were reported earlier for n-doped CdSe QDs.^74^ The relative contribution of τ_HET_ decreases with successive ZnS coating, which can be explained in terms of stronger confinement of the excited electrons in the core. Finally, *a*_3_ (Table S4) for the slowest time component (>1 ns) increased significantly with consecutive coating. We expect that this derives from the charge carrier recombination (τ_CCR_) to the ground state, as some carriers have nanosecond lifetime, and their population is increased by the addition of the ZnS shell.^67^

**Figure 5:**
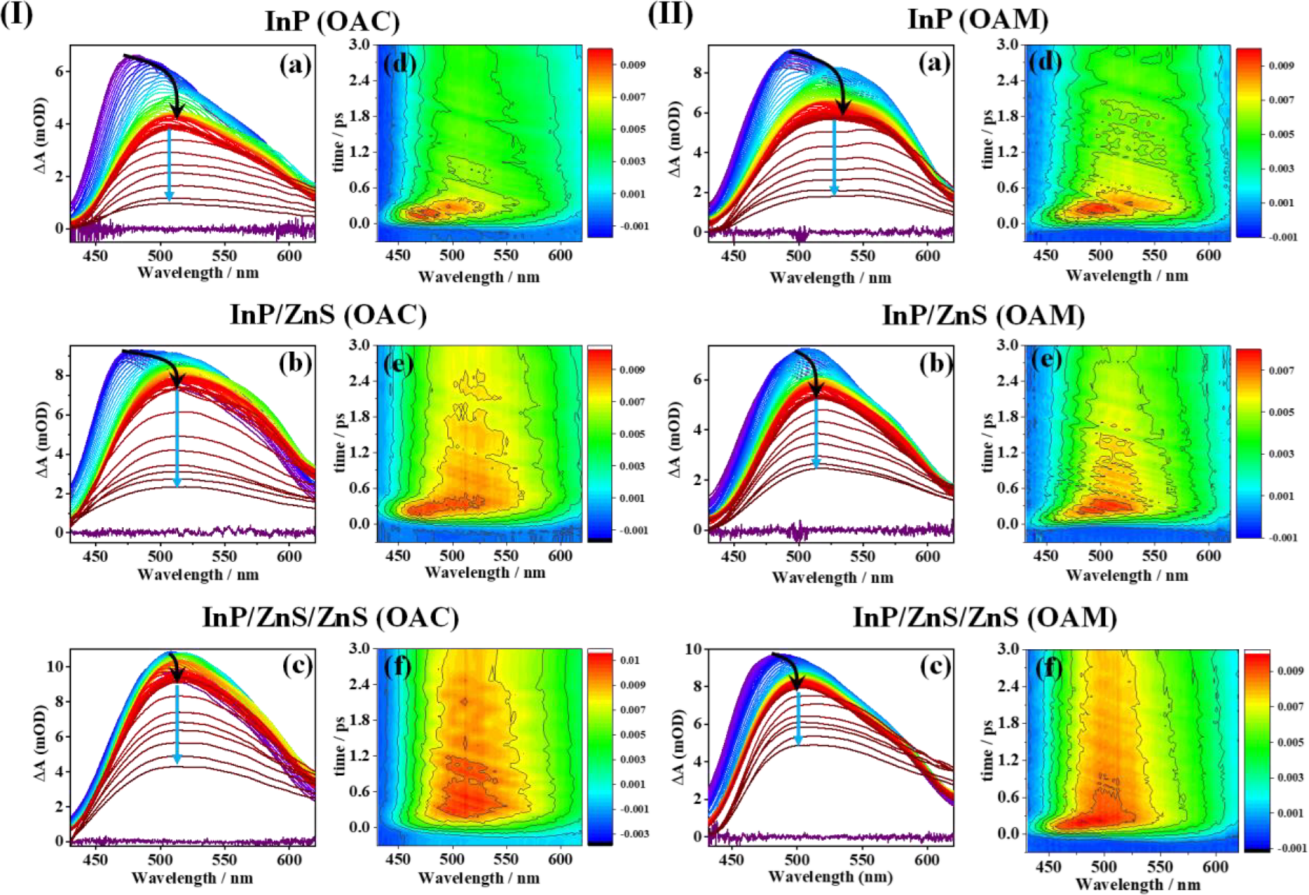
(I) Transient absorption spectra (TAS) of (a) InP, (b) InP/ZnS, and (c) InP/ZnS/ZnS QDs using OAC as surface ligand upon 340 nm excitation. Two-dimensional contour maps of the early time (0–3 ps) TA spectra of the same (d) InP, (e) InP/ZnS, and (f) InP/ZnS/ZnS QDs pumped at 340 nm. (II) TAS of the (a) InP, (b) InP/ZnS, and (c) InP/ZnS/ZnS QDs using OAM as surface ligand upon photoexcitation at 340 nm. Two-dimensional contour maps of the early time (0–3 ps) TAS of the same (d) InP, (e) InP/ZnS, and (f) InP/ZnS/ZnS QDs pumped at 340 nm. For the contour diagram, the color scales were normalized to the minimum/maximum ΔA values. Black line corresponds to delay 0–5 ps, and blue line corresponds to 5–500 ps.

Next, we probed the visible region of the transient signal of the QDs. Figure 5 shows the time evolution of the transient absorption spectra (TAS) and the 2D-contour plots for all the studied systems in the visible spectral range (430–620 nm). Upon excitation at 340 nm (energy ∼3.65 eV), a strong and positive excited state absorption signal was observed. Using the absorption cross-section and pump fluence, we estimated that the average number of excitons generated per QD, ⟨*N*⟩, to be lower than 1 (∼0.8) (SN2). However, multiple exciton generation with similar ⟨*N*⟩ values has been reported due to the presence of trap states.^75−78^ After excitation, the bare InP QDs with OAC and OAM surface ligands exhibited a positive band centered at 480 nm that appears within the IRF (∼160 fs). This band decayed within the first 2 ps, and a new band centered at 510 nm simultaneously appeared along with a shoulder at 580 nm. The TAS then decayed to a constant residual contribution in >1 ns. The intensity of the three bands differed slightly for the two uncoated QDs, indicating subtle changes in the ultrafast dynamics of the photogenerated charge carriers due to the differential electron donor ability of the employed ligand OAC and OAM.^36,37^ The TAS of the ZnS-coated QDs were similar to those of the bare QDs except that the contribution of the band at 480 nm to the overall spectra became less prevalent. The time evolution of the TAS within the first 2 ps suggests almost instantaneous trapping of the photoproduced charge carriers. Since we are working in the multiexciton regime, we expect that at least two electron–hole pairs can be formed simultaneously in the same QD.^62,73^ After the initial instantaneous trapping, the Auger process comes into play, which involves a rapid transfer of excess electronic energy to the holes that relax through the dense spectrum of VB states and get trapped in the surface trap states resulting in photoinduced absorption at longer wavelengths (510 and 580 nm).^67^

**Figure 6:**
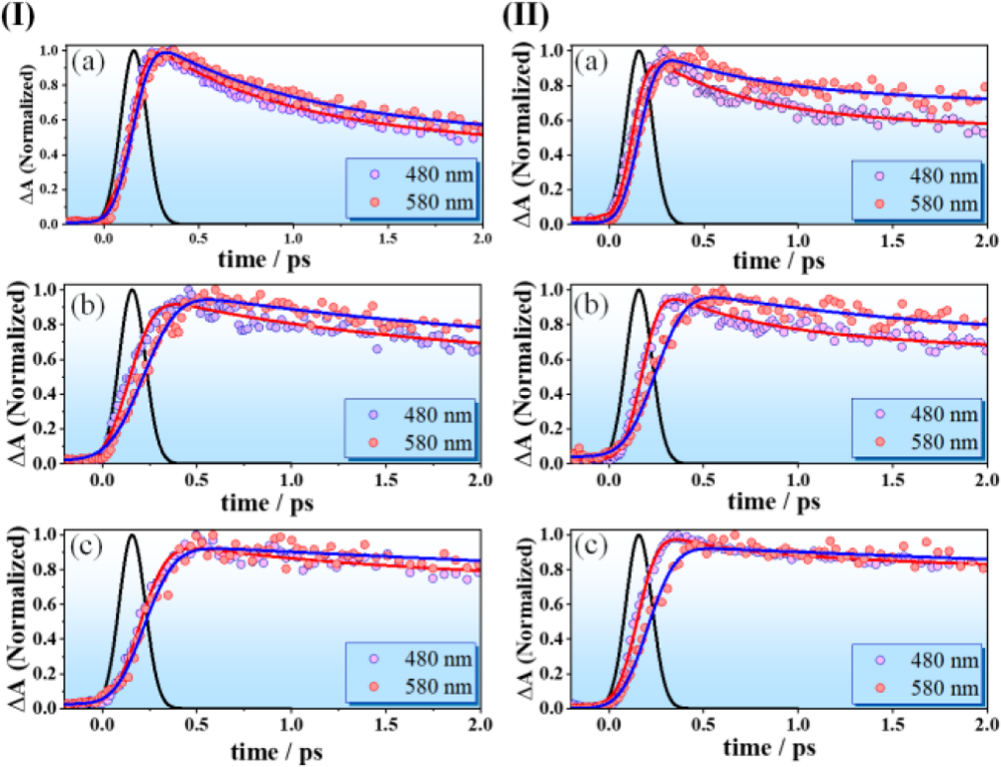
(I) Early time TA dynamics probed at 480 nm and 580 nm upon 340 nm excitation of (a) InP, (b) InP/ZnS, and (c) InP/ZnS/ZnS QDs with OAC as surface ligand. (II) Early time TA dynamics probed at 480 nm and 580 nm upon 340 nm excitation of (a) InP, (b) InP/ZnS, and (c) InP/ZnS/ZnS QDs with OAM as surface ligand.

To better understand the ultrafast dynamics of the nonradiative processes in these systems, we analyzed the transient decays at two representative probe wavelengths (480 and 580 nm). Upon band edge excitation, the positive photoinduced absorption signal corresponds predominantly to the hole dynamics.^75,79,80^ Thus, in the accessible TA spectral range for the blue emissive InP QDs in this study, we probe mainly the nonradiative processes associated with the relaxation of the photoproduced holes. At 510 nm, we expect a substantial overlap with the other bands (480 and 580 nm), which interferes with obtaining reliable parameters. Instead, the TAS were probed at 480 and 580 nm for the uncoated and coated QDs and were fitted by multiexponential functions (Figure 6, Figure S10, and Table S5). To begin with, for the bare InP QDs with both surface ligands, we got three components, a fast one with value of 1–1.5 ps, a comparatively slower one of 12–18 ps, and a longer one with values between 600 and 900 ps, independent of the probe wavelength. On the other hand, the transients for the coated InP QDs showed a different behavior at 480 and 580 nm. At 480 nm, we observed an ultrafast decay of 0.3 ps for the InP/ZnS QDs (for both ligands), which increased to 0.5 ps when the second shell was added (i.e., InP/ZnS/ZnS QDs). At this wavelength, we did not observe the ps-component that is present in the decays of the bare QDs. At 580 nm, the fit gave a rising component of 0.2 and 0.3 ps for the single and double ZnS coated InP QDs, respectively, independent of the used ligand. This ultrafast rising component was followed by a decay of 2 and 4 ps for single- and double-coated QDs, respectively. Similar to the bare QDs (12–15 ps), we also observed a longer time constant for the coated QDs. The value of this component increased with each consecutive ZnS coating to 30 and 90 ps (480 nm) and to 35 and 140 ps (580 nm) for InP QDs with OAC ligand having one and two ZnS shells, respectively. For the InP QDs with OAM, these values were 35 and 120 (480 nm), and 42 and 165 ps (580 nm), respectively. Finally, a third long-lived component was observed for the decays of all QDs, which for the bare InP QDs was 0.6–0.8 ns and increased to >1 ns after coating with ZnS. This trend was consistent with both surface ligands.

**Figure 7:**
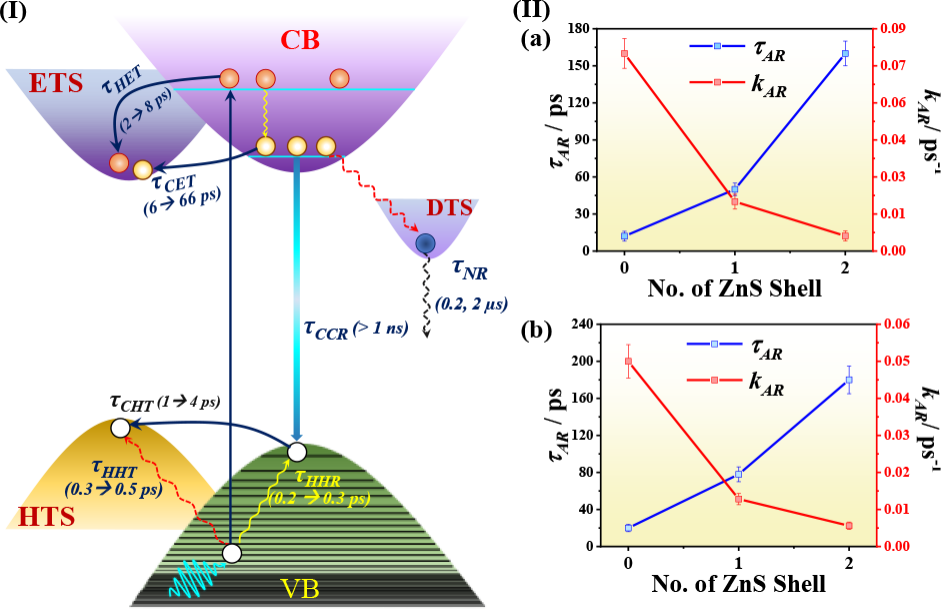
(I) Schematic mechanism diagram of hot carrier cooling for the blue emissive InP QDs. The changes in the time components upon ZnS coating are shown between the parentheses. (II) Variation of Auger recombination time and rates for InP QDs upon consecutive ZnS coating with (a) OAC and (b) OAM surface ligands. In the figure, ETS, HTS, CB, VB, and DTS stand for electron trap states, hole trap states, conduction band, valence band, and deep trap states, respectively.

Figure 7I and Table 4 present an overview of the observed time constants and the related processes. To assign the time components associated with the nonradiative relaxation of the photoproduced carriers in the blue emissive InP QDs, we first analyzed the subpicosecond decay probing at 480 nm for the coated QDs. Short-lived excited state species occurring in the first 2 ps can be explained in terms of the biexcitonic interaction between the band-edge electrons and hot excitons created by the pump.^62,81,82^ Since we are probing mainly the dynamics of the photoproduced holes in this spectral region, we assign the 300–500 fs time component to hot hole trapping (τ_HHT_).^22,66,56^ The value of the rising component at 580 nm is slightly lower than the decay values at 480 nm, which suggests the involvement of an additional process. Hot hole relaxation in 100–200 fs has been previously reported for red emissive InP QDs.^22,26,66^ Thus, we assign this rising component, observed at 580 nm, to the hot hole relaxation (τ_HHR_) via phonon scattering. Importantly, the decays at 480 and 580 nm for the uncoated InP QDs did not show any subpicosecond components, which we explain in terms of an ultrafast (<100 fs) hot hole trapping and relaxation that is below the IRF of our system.

**Table 4:** Charge Carrier Relaxation Time Constants for Different Processes Obtained from Fitting the Transient Decays at 920 nm in the IR Region and at 480 and 580 nm in the Visible Region upon Excitation at 340 nm^a^

ligand	QD	τ_HET_ (ps)	τ_CHT_ (ps)	τ_AR_ (ps)	τ_CCR_ (ns)	τ_HHT_ (ps)	τ_HHR_ (ps)
OAC	InP	2	1	15	>1	-	-
	InP/ZnS	5	2	35	>1	0.30	0.20
	InP/ZnS/ZnS	8	3.5	140	>1	0.50	0.30
OAM	InP	2	1	18	>1	-	-
	InP/ZnS	5	2	42	>1	0.30	0.20
	InP/ZnS/ZnS	8	4	165	>1	0.50	0.30

a The data are within 10–15% error limit.

This initial dynamics is followed by a process associated with the trapping of cold holes (τ_CHT_), which in green and red emissive InP and other QDs, was reported to occur in 1–5 ps scale.^22,40,66^ In our studies, for the bare QDs, the cold hole trapping occurred in 1–1.5 ps and was detected at both 480 and 580 nm, while for the ZnS-coated ones it was detectable only at 580 nm with time constant of 2–4 ps. The observed τ_CHT_ values are in agreement with those obtained from the ultrafast fluorescence experiments (0.5–9 ps). We assign the long ps-decay component obtained from the fs-TA traces at 580 nm to trap-assisted Auger recombination (τ_AR_). Similar behavior was observed in the decay traces collected at 480 nm but with slightly lower values for the time component. This long-time dynamic is associated with the decay of the more dominant 510 nm band at longer time delays. Trap-assisted Auger-like recombination is based on electron–hole Coulomb interaction and involves a rapid transfer of excess electronic energy to the trapped hole, which may quickly relax through the dense spectrum of VB states.^51,83^ Due to the abundance of trap states in the bare InP QDs, the trap-assisted Auger recombination is expected to be faster considering the increased overlap between the electron and hole wave functions and the reduction in momentum conservation requirements due to the spatial localization of such states.^75^ The fast Auger lifetime in the uncoated InP QDs can therefore be interpreted in terms of the “universal volume scaling law”, where the Auger recombination rate follows an inverse *R*^3^ dependence regardless of the chemical nature of the quantum dots.^22,84,85^ For the bare InP QDs with OAC and OAM surface ligands, τ_AR_ is 15 and 18 ps. However, successive ZnS coating increased the τ_AR_ values to 140 and 165 ps for the QDs with two ZnS layers, along with a significant decrease in the relative amplitudes (Table 4), which can be correlated directly to the passivation of the trap states.^26^ The decreased rate of biexciton Auger recombination (Figure 7IIa,b, Table S6) is comparable with similar results for quasi type-II QDs.^44,85,86^ This observation also confirms that for blue emissive InP QDs where the energy difference between the core and shell is not significant enough, quasi type-II nature may be present along with type-I characteristics (Figure 6b,c). In this regard, it is worth mentioning that a recent work has stated that the “universal volume scaling law” does not apply to core/shell QDs,^87^ while others support the idea that it is dependent on the type of the core/shell structure (type-I versus quasi type-II).^85^ Thus, the underlying mechanism for the increase in the values of the Auger recombination lifetimes by the addition of shells is more complex and could go beyond the universal volume scaling law. Finally, the third decay time (>1 ns) can be ascribed to the charge carrier recombination (τ_CCR_) of the holes and electrons and has already been discussed in the previous sections (Figure S10).^67^

### Microsecond Observation

**Figure 8:**
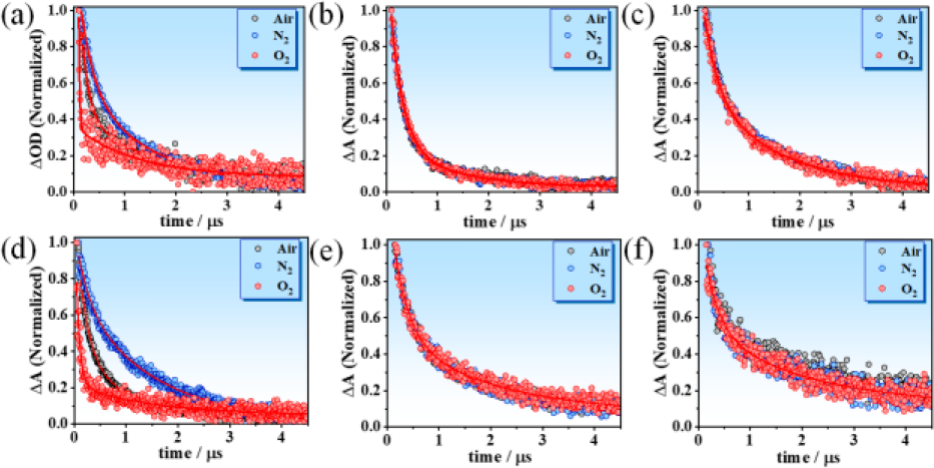
Normalized flash photolysis TA decays at 450 nm for (a, d) InP, (b, e) InP/ZnS, and (c, f) InP/ZnS/ZnS QDs with OAC and OAM as surface ligands, respectively, upon excitation at 355 nm.

Since the quantum yields of the uncoated and coated InP QDs do not exceed 45% and the maximum emission lifetime does not go beyond 9 ns, we suspect the presence of nonemitting defect states. Generally, the nonemitting defect states below the emitting trap states entrap the charges to promote competitive nonradiative pathways.^88^ Thus, to understand the nature of the photogenerated nonradiative long-lived deactivation channels, we performed laser flash photolysis experiments by irradiating the uncoated and coated InP QDs with 355 nm laser light in ambient conditions and in the presence of O_2_ and N_2_ (Figure 8). The decays were well-fitted by a biexponential function (Table S7). The two time components for the bare InP QDs at ambient conditions were 0.14 μs (72%) and 1.46 μs (28%), and 0.18 μs (71%) and 1.54 μs (29%) for OAM and OAC ligands, respectively. In an O_2_-rich atmosphere, the values of the short component were significantly reduced (from 0.14/0.18 to 0.02 μs for both ligands) with a concomitant increase in their relative contribution (from ∼70% to 90%). On the other hand, the values of the longer lifetime decreased only moderately from ∼1.5 μs in ambient conditions to ∼1.2 μs in an O_2_-rich atmosphere. The effect of the N_2_ atmosphere is also visible for the first component, while the longer one remains essentially unchanged. The first component increased from 0.14 to 0.26 μs for OAC and from 0.18 to 0.24 μs with OAM. Notably, while the effect of O_2_ and N_2_ was evident for the bare InP QDs, the ZnS-coated InP QDs did not reveal a change in the decay kinetics upon varying the atmosphere. We attribute the first component to deep surface electron trap states whereas the longer one to deep surface hole traps as they are mostly unaffected by N_2_ and O_2_. The effect of N_2_ and O_2_ on the deep electron traps can be observed only for the uncoated InP QDs. Therefore, we suggest that the molecular oxygen interacts with the surface defects in the bare QDs giving rise to the observed decrease in the value of the first component. Oxygen can accept the electrons from nonradiative surface trap levels decreasing the population of trapped electrons.^89,90^ On the other hand, the N_2_ effectively prevents the photo-oxidizing events on the surface of the bare particles giving values for deep electron traps that are similar to those for the ZnS-coated QDs. Thus, we suggest that the addition of the ZnS shell has a double positive effect; on one hand it passivates the surface traps present in the bare InP QDs, while on the other it protects the core from the photo-oxidation process with the molecular oxygen present in the atmosphere. However, it should be noted that these data preferentially delineate the nonradiative deep trap states below the emissive states that cause a significant decrease in the quantum yield values. These results suggest that developing further strategies to efficiently access the photogenerated carriers that reside at these deep trap states will not only increase the quantum yield but also drive forward the design of more efficient blue emissive QDs.

## Conclusions

In summary, we have demonstrated a convenient synthetic procedure for producing deep blue emissive ultrasmall InP QDs by utilizing two different surface ligands and using the less toxic and cost-effective tris(dimethylamino)phosphine. The ultrasmall InP QDs exhibited average sizes of 1.74 and 1.81 nm with oleic acid and oleylamine, respectively. The QDs have been successfully characterized by XRD, XPS, and HRTEM to determine their purity and crystallinity. Successive coating with a thin ZnS shell resulted in increments in the PLQY as high as 37% and 45%, respectively, with emission maxima at 410 and 430 nm, which are, to the best of our knowledge, the shortest emission wavelengths compared with those reported so far in the literature. The final coated QDs exhibited sizes of 4.11 and 4.15 nm with surface ligand OAC and OAM, respectively. Combining picosecond time-resolved measurements, femtosecond fluorescence up-conversion, and transient absorption measurements, we unravel the photophysical behavior by deciphering the charge carrier dynamics of the deep blue emissive InP QDs. The results reveal that the bare InP QDs with low PLQY undergo ultrafast electron and hole trapping along with efficient Auger recombination. However, the significant increase in the PLQY upon successive surface passivation with ZnS shells is found to be associated with slower hot electron and hole relaxation times. ZnS coating effectively enhances the lifetimes of the hot electrons from 2 to 8 ps and of the holes from subpicosecond to 0.5 ps. Surface passivation with ZnS also demonstrated increments of the lifetimes of the relaxed electrons and holes from 6 to 66 ps and from 1 to 4 ps, respectively. The rates for the trap-assisted Auger recombination decreased by an order of magnitude following coating with two ZnS shells. Knowledge of the nonemitting and relatively long-lived deep energy levels, which constitute additional competitive deactivation channels to the photoluminescence, has also been acquired with the help of laser flash photolysis spectroscopy. Therefore, the results highlight the effect of ZnS coating on electron and hole dynamics which may hold the key thereof for the rational design of highly efficient blue emissive LEDs composed of Cd- and Pb-free InP QDs.

## Supporting Information Available

Experimental Section; XPS characterization; time resolved PL decay; femtosecond emission transient decays; estimation of average exciton occupancies; short scale femtosecond emission transient decays; femtosecond transient decays at NIR region (PDF)

## References

[ref1] García de Arquer F. P.; Talapin D. V.; Klimov V. I.; Arakawa Y.; Bayer M.; Sargent E. H. Semiconductor Quantum Dots: Technological Progress and Future Challenges. Science 2021, 373, 640 10.1126/science.aaz8541. 34353926

[ref2] Melnychuk C.; Guyot-Sionnest P. Multicarrier Dynamics in Quantum Dots. Chem. Rev. 2021, 121, 2325–2372. 10.1021/acs.chemrev.0c00931. 33428388

[ref3] Carey G. H.; Abdelhady A. L.; Ning Z.; Thon S. M.; Bakr O. M.; Sargent E. H. Colloidal Quantum Dot Solar Cells. Chem. Rev. 2015, 115, 12732–12763. 10.1021/acs.chemrev.5b00063. 26106908

[ref4] Liu Z.; Lin C. H.; Hyun B. R.; Sher C. W.; Lv Z.; Luo B.; Jiang F.; Wu T.; Ho C. H.; Kuo H. C.; He J. H. Micro-Light-Emitting Diodes with Quantum Dots in Display Technology. Light Sci. Appl. 2020, 9, 83 10.1038/s41377-020-0268-1. 32411368 PMC7214519

[ref5] Kagan C. R.; Lifshitz E.; Sargent E. H.; Talapin D. V. Building Devices from Colloidal Quantum Dots. Science 2016, 353, 6302 10.1126/science.aac5523. 27563099

[ref6] Jang E.; Kim Y.; Won Y. H.; Jang H.; Choi S. M. Environmentally Friendly InP-Based Quantum Dots for Efficient Wide Color Gamut Displays. ACS Energy Lett. 2020, 5 (4), 1316–1327. 10.1021/acsenergylett.9b02851.

[ref7] El-Ballouli A. O.; Bakr O. M.; Mohammed O. F. Compositional, Processing, and Interfacial Engineering of Nanocrystal- And Quantum-Dot-Based Perovskite Solar Cells. Chem. Mater. 2019, 31, 6387–6411. 10.1021/acs.chemmater.9b01268.

[ref8] El-Ballouli A. O.; Alarousu E.; Bernardi M.; Aly S. M.; Lagrow A. P.; Bakr O. M.; Mohammed O. F. Quantum Confinement-Tunable Ultrafast Charge Transfer at the PbS Quantum Dot and Phenyl-C61-Butyric Acid Methyl Ester Interface. J. Am. Chem. Soc. 2014, 136, 6952–6959. 10.1021/ja413254g. 24521255

[ref9] Rakshit S.; Piatkowski P.; Mora-Seró I.; Douhal A. Combining Perovskites and Quantum Dots: Synthesis, Characterization, and Applications in Solar Cells, LEDs, and Photodetectors. Adv. Opt. Mater. 2022, 10, 2102566 10.1002/adom.202102566.

[ref10] Xu G.; Zeng S.; Zhang B.; Swihart M. T.; Yong K. T.; Prasad P. N. New Generation Cadmium-Free Quantum Dots for Biophotonics and Nanomedicine. Chem. Rev. 2016, 116, 12234–12327. 10.1021/acs.chemrev.6b00290. 27657177

[ref11] Chen B.; Li D.; Wang F. InP Quantum Dots: Synthesis and Lighting Applications. Small 2020, 16, 2002454 10.1002/smll.202002454. 32613755

[ref12] Won Y. H.; Cho O.; Kim T.; Chung D. Y.; Kim T.; Chung H.; Jang H.; Lee J.; Kim D.; Jang E. Highly Efficient and Stable InP/ZnSe/ZnS Quantum Dot Light-Emitting Diodes. Nature 2019, 575, 634–638. 10.1038/s41586-019-1771-5. 31776489

[ref13] Liu P.; Lou Y.; Ding S.; Zhang W.; Wu Z.; Yang H.; Xu B.; Wang K.; Sun X. W. Green InP/ZnSeS/ZnS Core Multi-Shelled Quantum Dots Synthesized with Aminophosphine for Effective Display Applications. Adv. Funct. Mater. 2021, 31, 2008453 10.1002/adfm.202008453.

[ref14] Zhang H.; Ma X.; Lin Q.; Zeng Z.; Wang H.; Li L. S.; Shen H.; Jia Y.; Du Z. High-Brightness Blue InP Quantum Dot-Based Electroluminescent Devices: The Role of Shell Thickness. J. Phys. Chem. Lett. 2020, 11, 960–967. 10.1021/acs.jpclett.9b03567. 31957438

[ref15] Zhang W.; Tan Y.; Duan X.; Zhao F.; Liu H.; Chen W.; Liu P.; Liu X.; Wang K.; Zhang Z.; Sun X. W. High Quantum Yield Blue InP/ZnS/ZnS Quantum Dots Based on Bromine Passivation for Efficient Blue Light-Emitting Diodes. Adv. Opt. Mater. 2022, 10, 2200685 10.1002/adom.202200685.

[ref16] Wang S.; Yu J.; Ye H.; Chi M.; Yang H.; Wang H.; Cao F.; Li W.; Kong L.; Wang L.; Chen R.; Yang X. Low-Threshold Amplified Spontaneous Emission in Blue Quantum Dots Enabled by Effectively Suppressing Auger Recombination. Adv. Opt. Mater. 2021, 9, 2100068 10.1002/adom.202100068.

[ref17] Shen W.; Tang H.; Yang X.; Cao Z.; Cheng T.; Wang X.; Tan Z.; You J.; Deng Z. Synthesis of Highly Fluorescent InP/ZnS Small-Core/Thick-Shell Tetrahedral-Shaped Quantum Dots for Blue Light-Emitting Diodes. J. Mater. Chem. C 2017, 5, 8243–8249. 10.1039/C7TC02927F.

[ref18] Ramasamy P.; Kim N.; Kang Y. S.; Ramirez O.; Lee J. S. Tunable, Bright, and Narrow-Band Luminescence from Colloidal Indium Phosphide Quantum Dots. Chem. Mater. 2017, 29 (16), 6893–6899. 10.1021/acs.chemmater.7b02204.

[ref19] Zhang W.; Ding S.; Zhuang W.; Wu D.; Liu P.; Qu X.; Liu H.; Yang H.; Wu Z.; Wang K.; Sun X. W. InP/ZnS/ZnS Core/Shell Blue Quantum Dots for Efficient Light-Emitting Diodes. Adv. Funct. Mater. 2020, 30, 2005303 10.1002/adfm.202005303.

[ref20] Kwon Y.; Oh J.; Lee E.; Lee S. H.; Agnes A.; Bang G.; Kim J.; Kim D.; Kim S. Evolution from Unimolecular to Colloidal-Quantum-Dot-like Character in Chlorine or Zinc Incorporated InP Magic Size Clusters. Nat. Commun. 2020, 11, 3127 10.1038/s41467-020-16855-9. 32561721 PMC7305325

[ref21] Janke E. M.; Williams N. E.; She C.; Zherebetskyy D.; Hudson M. H.; Wang L.; Gosztola D. J.; Schaller R. D.; Lee B.; Sun C.; Engel G. S.; Talapin D. V. Origin of Broad Emission Spectra in InP Quantum Dots: Contributions from Structural and Electronic Disorder. J. Am. Chem. Soc. 2018, 140, 15791–15803. 10.1021/jacs.8b08753. 30285448

[ref22] Yang W.; Yang Y.; Kaledin A. L.; He S.; Jin T.; McBride J. R.; Lian T. Surface Passivation Extends Single and Biexciton Lifetimes of InP Quantum Dots. Chem. Sci. 2020, 11, 5779–5789. 10.1039/D0SC01039A. 32832054 PMC7416692

[ref23] Zhang X.; Hudson M. H.; Castellano F. N. Passivation of Electron Trap States in InP Quantum Dots with Benzoic Acid Ligands. J. Phys. Chem. C 2021, 125, 18362–18371. 10.1021/acs.jpcc.1c05594.

[ref24] Zhang X.; Hudson M. H.; Castellano F. N. Engineering Long-Lived Blue Photoluminescence from InP Quantum Dots Using Isomers of Naphthoic Acid. J. Am. Chem. Soc. 2022, 144, 3527–3534. 10.1021/jacs.1c12207. 35188779

[ref25] Zhang B.; Wang X.; Wang D.; Tang J.; Fang X.; Fang D.; Wang X.; Chen R.; He T.; Wei Z. Ultrafast Charge Carrier Dynamics and Nonlinear Optical Absorption of InP/ZnS Core-Shell Colloidal Quantum Dots. J. Phys. Chem. C 2019, 123, 27207–27213. 10.1021/acs.jpcc.9b07092.

[ref26] Park J.; Won Y. H.; Han Y.; Kim H. M.; Jang E.; Kim D. Tuning Hot Carrier Dynamics of InP/ZnSe/ZnS Quantum Dots by Shell Morphology Control. Small 2022, 18, 2105492 10.1002/smll.202105492. 34889031

[ref27] Hao J.; Liu H.; Miao J.; Lu R.; Zhou Z.; Zhao B.; Xie B.; Cheng J.; Wang K.; Delville M. H. A Facile Route to Synthesize CdSe/ZnS Thick-Shell Quantum Dots with Precisely Controlled Green Emission Properties: Towards QDs Based LED Applications. Sci. Rep. 2019, 9, 12048 10.1038/s41598-019-48469-7. 31427624 PMC6700096

[ref28] Jo J. H.; Jo D. Y.; Lee S. H.; Yoon S. Y.; Lim H. B.; Lee B. J.; Do Y. R.; Yang H. InP-Based Quantum Dots Having an InP Core, Composition-Gradient ZnSeS Inner Shell, and ZnS Outer Shell with Sharp, Bright Emissivity, and Blue Absorptivity for Display Devices. ACS Appl. Nano Mater. 2020, 3, 1972–1980. 10.1021/acsanm.0c00008.

[ref29] Virieux H.; Le Troedec M.; Cros-Gagneux A.; Ojo W. S.; Delpech F.; Nayral C.; Martinez H.; Chaudret B. InP/ZnS Nanocrystals: Coupling NMR and XPS for Fine Surface and Interface Description. J. Am. Chem. Soc. 2012, 134, 19701–19708. 10.1021/ja307124m. 23131073

[ref30] Nemoto K.; Watanabe J.; Sun H.; Shirahata N. Coherent InP/ZnS Core@shell Quantum Dots with Narrow-Band Green Emissions. Nanoscale 2022, 14, 9900–9909. 10.1039/D2NR02071H. 35781556

[ref31] Pu Y. C.; Fan H. C.; Chang J. C.; Chen Y. H.; Tseng S. W. Effects of Interfacial Oxidative Layer Removal on Charge Carrier Recombination Dynamics in InP/ZnSexS1- Xcore/Shell Quantum Dots. J. Phys. Chem. Lett. 2021, 12, 7194–7200. 10.1021/acs.jpclett.1c02125. 34309384

[ref32] Toufanian R.; Chern M.; Kong V. H.; Dennis A. M. Engineering Brightness-Matched Indium Phosphide Quantum Dots. Chem. Mater. 2021, 33, 1964–1975. 10.1021/acs.chemmater.0c03181. 34219920 PMC8243842

[ref33] Selopal G. S.; Zhao H.; Wang Z. M.; Rosei F. Core/Shell Quantum Dots Solar Cells. Adv. Funct. Mater. 2020, 30, 1908762 10.1002/adfm.201908762.

[ref34] Saha A.; Konstantatos G. Ag2ZnSnS4-ZnS Core-Shell Colloidal Quantum Dots: A near-Infrared Luminescent Material Based on Environmentally Friendly Elements. J. Mater. Chem. C 2021, 9, 5682–5688. 10.1039/D1TC00421B. PMC8101413 33996096

[ref35] Heuer-Jungemann A.; Feliu N.; Bakaimi I.; Hamaly M.; Alkilany A.; Chakraborty I.; Masood A.; Casula M. F.; Kostopoulou A.; Oh E.; Susumu K.; Stewart M. H.; Medintz I. L.; Stratakis E.; Parak W. J.; Kanaras A. G. The Role of Ligands in the Chemical Synthesis and Applications of Inorganic Nanoparticles. Chem. Rev. 2019, 119, 4819–4880. 10.1021/acs.chemrev.8b00733. 30920815

[ref36] Zhang H.; Jing L.; Zeng J.; Hou Y.; Li Z.; Gao M. Revisiting the Coordination Chemistry for Preparing Manganese Oxide Nanocrystals in the Presence of Oleylamine and Oleic Acid. Nanoscale 2014, 6 (11), 5918–5925. 10.1039/c4nr00761a. 24760344

[ref37] Peng Y.; Lu B.; Wang N.; Li L.; Chen S. Impacts of Interfacial Charge Transfer on Nanoparticle Electrocatalytic Activity towards Oxygen Reduction. Phys. Chem. Chem. Phys. 2017, 19 (14), 9336–9348. 10.1039/C6CP08925A. 28165087

[ref38] Kim T. G.; Zherebetskyy D.; Bekenstein Y.; Oh M. H.; Wang L. W.; Jang E.; Alivisatos A. P. Trap Passivation in Indium-Based Quantum Dots through Surface Fluorination: Mechanism and Applications. ACS Nano 2018, 12 (11), 11529–11540. 10.1021/acsnano.8b06692. 30335943

[ref39] Jin S.; Harris R. D.; Lau B.; Aruda K. O.; Amin V. A.; Weiss E. A. Enhanced Rate of Radiative Decay in Cdse Quantum Dots upon Adsorption of an Exciton-Delocalizing Ligand. Nano Lett. 2014, 14 (9), 5323–5328. 10.1021/nl5023699. 25167466

[ref40] Eagle F. W.; Park N.; Cash M.; Cossairt B. M. Surface Chemistry and Quantum Dot Luminescence: Shell Growth, Atomistic Modification, and Beyond. ACS Energy Lett. 2021, 6 (3), 977–984. 10.1021/acsenergylett.0c02697.

[ref41] Mićić O. I.; Cheong H. M.; Fu H.; Zunger A.; Sprague J. R.; Mascarenhas A.; Nozik A. J. Size-Dependent Spectroscopy of InP Quantum Dots. J. Phys. Chem. B 1997, 101, 4904–4912. 10.1021/jp9704731.

[ref42] Reid K. R.; McBride J. R.; Freymeyer N. J.; Thal L. B.; Rosenthal S. J. Chemical Structure, Ensemble and Single-Particle Spectroscopy of Thick-Shell InP-ZnSe Quantum Dots. Nano Lett. 2018, 18, 709–716. 10.1021/acs.nanolett.7b03703. 29282985 PMC6163126

[ref43] Kim T. G.; Zherebetskyy D.; Bekenstein Y.; Oh M. H.; Wang L. W.; Jang E.; Alivisatos A. P. Trap Passivation in Indium-Based Quantum Dots through Surface Fluorination: Mechanism and Applications. ACS Nano 2018, 12, 11529–11540. 10.1021/acsnano.8b06692. 30335943

[ref44] Park Y. S.; Bae W. K.; Baker T.; Lim J.; Klimov V. I. Effect of Auger Recombination on Lasing in Heterostructured Quantum Dots with Engineered Core/Shell Interfaces. Nano Lett. 2015, 15 (11), 7319–7328. 10.1021/acs.nanolett.5b02595. 26397312

[ref45] Cohn A. W.; Schimpf A. M.; Gunthardt C. E.; Gamelin D. R. Size-Dependent Trap-Assisted Auger Recombination in Semiconductor Nanocrystals. Nano Lett. 2013, 13, 1810–1815. 10.1021/nl400503s. 23464673

[ref46] Lee Y.; Jo D.-Y.; Kim T.; Jo J.-H.; Park J.; Yang H.; Kim D. Effectual Interface and Defect Engineering for Auger Recombination Suppression in Bright InP/ZnSeS/ZnS Quantum Dots. ACS Appl. Mater. Interfaces 2022, 14, 12479–12487. 10.1021/acsami.1c20088. 35238532

[ref47] Hinuma Y.; Grüneis A.; Kresse G.; Oba F. Band Alignment of Semiconductors from Density-Functional Theory and Many-Body Perturbation Theory. Phys. Rev. B: Condens. Matter Mater. Phys. 2014, 90, 155405 10.1103/PhysRevB.90.155405.

[ref48] García-Santamaría F.; Brovelli S.; Viswanatha R.; Hollingsworth J. a; Htoon H.; Crooker S. a; Klimov V. I. Heteronanocrystals: The Role of the Core - Shell Interface. Nano Lett. 2011, 11, 687–693. 10.1021/nl103801e. 21207930

[ref49] Pandey A.; Guyot-Sionnest P. Slow Electron Cooling in Colloidal Quantum Dots. Science 2008, 322, 929–932. 10.1126/science.1159832. 18988849

[ref50] Spoor F. C. M.; Kunneman L. T.; Evers W. H.; Renaud N.; Grozema F. C.; Houtepen A. J.; Siebbeles L. D. A. Hole Cooling Is Much Faster than Electron. ACS Nano 2016, 10, 695–703. 10.1021/acsnano.5b05731. 26654878

[ref51] Blackburn J. L.; Ellingson R. J.; Mićić O. I.; Nozik A. J. Electron Relaxation in Colloidal InP Quantum Dots with Photogenerated Excitons or Chemically Injected Electrons. J. Phys. Chem. B 2003, 107, 102–109. 10.1021/jp026746w.

[ref52] Rodosthenous P.; Gómez-Campos F. M.; Califano M. Tuning the Radiative Lifetime in InP Colloidal Quantum Dots by Controlling the Surface Stoichiometry. J. Phys. Chem. Lett. 2020, 11, 10124–10130. 10.1021/acs.jpclett.0c02752. 33191752

[ref53] Mićić O. I.; Nozik A. J.; Lifshitz E.; Rajh T.; Poluektov O. G.; Thurnauer M. C. Electron and Hole Adducts Formed in Illuminated InP Colloidal Quantum Dots Studied by Electron Paramagnetic Resonance. J. Phys. Chem. B 2002, 106, 4390–4395. 10.1021/jp014180q.

[ref54] Houtepen A. J.; Hens Z.; Owen J. S.; Infante I. On the Origin of Surface Traps in Colloidal II-VI Semiconductor Nanocrystals. Chem. Mater. 2017, 29, 752–761. 10.1021/acs.chemmater.6b04648.

[ref55] Mourdikoudis S.; Liz-Marzán L. M. Oleylamine in Nanoparticle Synthesis. Chem. Mater. 2013, 25, 1465–1476. 10.1021/cm4000476.

[ref56] Freymeyer N. J.; Click S. M.; Reid K. R.; Chisholm M. F.; Bradsher C. E.; McBride J. R.; Rosenthal S. J. Effect of Indium Alloying on the Charge Carrier Dynamics of Thick-Shell InP/ZnSe Quantum Dots. J. Chem. Phys. 2020, 152, 161104 10.1063/1.5145189. 32357779

[ref57] Keene J. D.; McBride J. R.; Orfield N. J.; Rosenthal S. J. Elimination of Hole-Surface Overlap in Graded CdSxSe1- x Nanocrystals Revealed by Ultrafast Fluorescence Upconversion Spectroscopy. ACS Nano 2014, 8, 10665–10673. 10.1021/nn504235w. 25203834

[ref58] Underwood D. F.; Kippeny T.; Rosenthal S. J. Ultrafast Carrier Dynamics in CdSe Nanocrystals Determined by Femtosecond Fluorescence Upconversion Spectroscopy. J. Phys. Chem. B 2001, 105, 436–443. 10.1021/jp003088b.

[ref59] Klimov V.; Bolivar P. H.; Kurz H. Ultrafast Carrier Dynamics in Semiconductor Quantum Dots. Phys. Rev. B - Condens. Matter Mater. Phys. 1996, 53, 1463–1467. 10.1103/PhysRevB.53.1463. 9983607

[ref60] Kim Y.; Chang J. H.; Choi H.; Kim Y. H.; Bae W. K.; Jeong S. III-V Colloidal Nanocrystals: Control of Covalent Surfaces. Chem. Sci. 2020, 11, 913–922. 10.1039/C9SC04290C. PMC8145357 34084346

[ref61] Knowles K. E.; McArthur E. A.; Weiss E. A. A Multi-Timescale Map of Radiative and Nonradiative Decay Pathways for Excitons in CdSe Quantum Dots. ACS Nano 2011, 5, 2026–2035. 10.1021/nn2002689. 21361353

[ref62] Das S.; Rakshit S.; Datta A. Interplay of Multiexciton Relaxation and Carrier Trapping in Photoluminescent CdS Quantum Dots Prepared in Aqueous Medium. J. Phys. Chem. C 2020, 124, 28313–28322. 10.1021/acs.jpcc.0c09366.

[ref63] Brodu A.; Ballottin M. V.; Buhot J.; Dupont D.; Tessier M.; Hens Z.; Rabouw F. T.; Christianen P. C. M.; De Mello Donega C.; Vanmaekelbergh D. Exciton-Phonon Coupling in InP Quantum Dots with ZnS and (Zn,Cd) Se Shells. Phys. Rev. B 2020, 101, 125413 10.1103/PhysRevB.101.125413.

[ref64] Lad A. D.; Mahamuni S. Effect of ZnS Shell Formation on the Confined Energy Levels of ZnSe Quantum Dots. Phys. Rev. B: Condens. Matter Mater. Phys. 2008, 78, 125421 10.1103/PhysRevB.78.125421.

[ref65] Rainò G.; Stöferle T.; Moreels I.; Gomes R.; Kamal J. S.; Hens Z.; Mahrt R. F. Probing the Wave Function Delocalization in CdSe/CdS Dot-in-Rod Nanocrystals by Time-and Temperature-Resolved Spectroscopy. ACS Nano 2011, 5 (5), 4031–4036. 10.1021/nn2005969. 21504193

[ref66] Richter A. F.; Binder M.; Bohn B. J.; Grumbach N.; Neyshtadt S.; Urban A. S.; Feldmann J. Fast Electron and Slow Hole Relaxation in InP-Based Colloidal Quantum Dots. ACS Nano 2019, 13, 14408–14415. 10.1021/acsnano.9b07969. 31790203

[ref67] McArthur E. A.; Morris-Cohen A. J.; Knowles K. E.; Weiss E. A. Charge Carrier Resolved Relaxation of the First Excitonic State in CdSe Quantum Dots Probed with Near-Infrared Transient Absorption Spectroscopy. J. Phys. Chem. B 2010, 114 (45), 14514–14520. 10.1021/jp102101f. 20507144

[ref68] Nozik A. J. Multiple Exciton Generation in Semiconductor Quantum Dots. Chem. Phys. Lett. 2008, 457 (1–3), 3–11. 10.1016/j.cplett.2008.03.094. 26295422

[ref69] Klimov V. I.; Schwarz C. J.; Mc Branch D. W.; Leatherdale C. A.; Bawendi M. G. Ultrafast Dynamics of Inter- and Intraband Transitions in Semiconductor Nanocrystals: Implications for Quantum-Dot Lasers. Phys. Rev. B: Condens. Matter Mater. Phys. 1999, 60 (4), R2177–R2180. 10.1103/PhysRevB.60.R2177.

[ref70] Guyot-Sionnest P.; Shim M.; Matranga C.; Hines M. Intraband Relaxation in CdSe Quantum Dots. Phys. Rev. B - Condens. Matter Mater. Phys. 1999, 60 (4), R2181–R2184. 10.1103/PhysRevB.60.R2181.

[ref71] Piatkowski P.; Cohen B.; Javier Ramos F.; Di Nunzio M.; Nazeeruddin M. K.; Grätzel M.; Ahmad S.; Douhal A. Direct Monitoring of Ultrafast Electron and Hole Dynamics in Perovskite Solar Cells. Phys. Chem. Chem. Phys. 2015, 17 (22), 14674–14684. 10.1039/C5CP01119A. 25972103

[ref72] Kobosko S. M.; Kamat P. V. Indium-Rich AgInS2-ZnS Quantum Dots - Ag-/Zn-Dependent Photophysics and Photovoltaics. J. Phys. Chem. C 2018, 122, 14336–14344. 10.1021/acs.jpcc.8b03001.

[ref73] Eliasson N.; Rimgard B. P.; Castner A.; Tai C. W.; Ott S.; Tian H.; Hammarström L. Ultrafast Dynamics in Cu-Deficient CuInS2Quantum Dots: Sub-Bandgap Transitions and Self-Assembled Molecular Catalysts. J. Phys. Chem. C 2021, 125, 14751–14764. 10.1021/acs.jpcc.1c02468.

[ref74] Wang J.; Wang L.; Yu S.; Ding T.; Xiang D.; Wu K. Spin Blockade and Phonon Bottleneck for Hot Electron Relaxation Observed in N-Doped Colloidal Quantum Dots. Nat. Commun. 2021, 12 (1), 550 10.1038/s41467-020-20835-4. 33483503 PMC7822822

[ref75] Kambhampati P. Hot Exciton Relaxation Dynamics in Semiconductor Quantum Dots: Radiationless Transitions on the Nanoscale. J. Phys. Chem. C 2011, 115, 22089–22109. 10.1021/jp2058673.

[ref76] Mcguire J. A.; Joo J.; Pietryga J. M.; Schaller R. D.; Klimov V. I. New Aspects of Carrier Multiplication in Semiconductor Nanocrystals. Acc. Chem. Res. 2008, 41, 1810–1819. 10.1021/ar800112v. 19006342

[ref77] Smith C. T.; Leontiadou M. A.; Clark P. C. J.; Lydon C.; Savjani N.; Spencer B. F.; Flavell W. R.; O’Brien P.; Binks D. J. Multiple Exciton Generation and Dynamics in InP/CdS Colloidal Quantum Dots. J. Phys. Chem. C 2017, 121, 2099–2107. 10.1021/acs.jpcc.6b11744.

[ref78] Beard M. C. Multiple Exciton Generation in Semiconductor Quantum Dots. J. Phys. Chem. Lett. 2011, 2, 1282–1288. 10.1021/jz200166y. 26295422

[ref79] Cooney R. R.; Sewall S. L.; Anderson K. E. H.; Dias E. A.; Kambhampati P. Breaking the Phonon Bottleneck for Holes in Semiconductor Quantum Dots. Phys. Rev. Lett. 2007, 98 (17), 177403 10.1103/PhysRevLett.98.177403.

[ref80] Kambhampati P. Unraveling the Structure and Dynamics of Excitons in Semiconductor Quantum Dots. Acc. Chem. Res. 2011, 44 (1), 1–13. 10.1021/ar1000428. 20942416

[ref81] Sewall S. L.; Cooney R. R.; Anderson K. E. H.; Dias E. A.; Sagar D. M.; Kambhampati P. State-Resolved Studies of Biexcitons and Surface Trapping Dynamics in Semiconductor Quantum Dots. J. Chem. Phys. 2008, 129, 084701 10.1063/1.2971181. 19044835

[ref82] Saari J. I.; Dias E. A.; Reifsnyder D.; Krause M. M.; Walsh B. R.; Murray C. B.; Kambhampati P. Ultrafast Electron Trapping at the Surface of Semiconductor Nanocrystals: Excitonic and Biexcitonic Processes. J. Phys. Chem. B 2013, 117, 4412–4421. 10.1021/jp307668g. 23186016

[ref83] Klimov V. I. Spectral and Dynamical Properties of Multiexcitons in Semiconductor Nanocrystals. Annu. Rev. Phys. Chem. 2007, 58, 635–673. 10.1146/annurev.physchem.58.032806.104537. 17163837

[ref84] Robel I.; Gresback R.; Kortshagen U.; Schaller R. D.; Klimov V. I. Universal Size-Dependent Trend in Auger Recombination in Direct-Gap and Indirect-Gap Semiconductor Nanocrystals. Phys. Rev. Lett. 2009, 102, 177404 10.1103/PhysRevLett.102.177404. 19518831

[ref85] Philbin J. P.; Rabani E. Auger Recombination Lifetime Scaling for Type i and Quasi-Type II Core/Shell Quantum Dots. J. Phys. Chem. Lett. 2020, 11, 5132–5138. 10.1021/acs.jpclett.0c01460. 32513003

[ref86] Hou X.; Li Y.; Qin H.; Peng X. Effects of Interface-Potential Smoothness and Wavefunction Delocalization on Auger Recombination in Colloidal CdSe-Based Core/Shell Quantum Dots. J. Chem. Phys. 2019, 151 (23), 234703 10.1063/1.5125940. 31864257

[ref87] Kong D.; Jia Y.; Ren Y.; Xie Z.; Wu K.; Lian T. Shell-Thickness-Dependent Biexciton Lifetime in Type i and Quasi-Type II CdSe@CdS Core/Shell Quantum Dots. J. Phys. Chem. C 2018, 122, 14091–14098. 10.1021/acs.jpcc.8b01234.

[ref88] Wojdyla M.; Gallagher S. A.; Moloney P.; Gun’ko Y. K.; Kelly J. M.; Magno L. M.; Quinn S. J.; Clark I. P.; Greetham G. M.; Towrie M. Picosecond to Millisecond Transient Absorption Spectroscopy of Broad-Band Emitting Chiral CdSe Quantum Dots. J. Phys. Chem. C 2012, 116, 16226–16232. 10.1021/jp3023088.

[ref89] Hu Z.; Liu S.; Qin H.; Zhou J.; Peng X. Oxygen Stabilizes Photoluminescence of CdSe/CdS Core/Shell Quantum Dots via Deionization. J. Am. Chem. Soc. 2020, 142, 4254–4264. 10.1021/jacs.9b11978. 32045520

[ref90] van Sark W. G. J. H. M.; Frederix P. L. T. M.; Bol A. A.; Gerritsen H. C.; Meijerink A. Blueing, Bleaching, and Blinking of Single CdSe/ZnS Quantum Dots. ChemPhysChem 2002, 3, 871–879. 10.1002/1439-7641(20021018)3:10<871::AID-CPHC871>3.0.CO;2-T.

