# Supplementary material for: Deep Blue and Highly Emissive ZnS-Passivated InP QDs: Facile Synthesis, Characterization, and Deciphering of Their Ultrafast-to-Slow Photodynamics
Source: ACS Appl Mater Interfaces. 2023 Jan 6;15(2):3099–111. doi: 10.1021/acsami.2c16289 (PMC10089568; doi:10.1021/acsami.2c16289)
Supplement: Supplementary file 1 — am2c16289_si_001.pdf [file am2c16289_si_001.pdf]

Supporting Information

Deep Blue and Highly Emissive ZnS-Passivated InP QDs: Facile Synthesis, Characterization and Deciphering their Ultrafast-to-Slow Photodynamics

Soumyadipta Rakshit,† Boiko Cohen,† Mario Gutiérrez,† Ala'a O. El-Ballouli,† and Abderrazzak Douhal*†

†Departamento de Química Física, Facultad de Ciencias Ambientales y Bioquímica and INAMOL, Universidad de Castilla-La Mancha, Avenida Carlos III, Toledo 45071, Spain

*Corresponding Author.
E-mail: Abderrazzak.douhal@uclm.es

Table of Contents

1.	S1	Experimental Section	S2-5
2.	SN1	Equation for calculating average radiative and non-radiative rate constants	S-5
3.	SN2	Calculation of average number of excitons	S5-6
4.	Figure S1	Size distribution histogram of InP core, InP/ZnS QDs and InP/ZnS/ZnS QDs in two different surface ligands obtained from TEM. Full XPS spectrum of the ultrasmall InP QDs and (III) InP/ZnS/ZnS QDs and high-resolution scan of each element	S-7
5.	Figure S2	Change in the absorption spectrum of InP QDs upon ZnS shell formation. Emission spectra of each material under the same experimental conditions	S-8
6.	Figure S3	Illustration of the emission color of InP core QDs with their respecitve sizes	S-9
7.	Scheme S2	Schematic representation of the energy bandgap in InP/ZnS/ZnS QDs	S-9
8.	Figure S4	Time resolved PL decays at different emission wavelengths	S-10
9.	Figure S5	Femtosecond emission transient decays of coated and uncoated InP QDs with different surface ligands at 425 nm probe wavelength	S-11
10.	Figure S6	Short window femtosecond emission transient decays of coated and uncoated InP QDs with different surface ligands at different probed wavelengths	S-12
11.	Figure S7	Femtosecond emission transient decays of coated and uncoated InP QDs with different surface ligands at different probed wavelengths	S-13
12.	Figure S8	Femtosecond transient absorption spectra of the QDs in the NIR region	S-14
13.	Figure S9	Femtosecond transient decays of coated and uncoated InP QDs with different surface ligands at 870 and 970 nm probed wavelengths	S-15
14.	Figure S10	Femtosecond transient decays of coated and uncoated InP QDs with different surface ligands at 480 and 580 nm probed wavelengths	S-16
15.	Table S1	InP core sizes and emission wavelength maxima reported previously and in the present work	S-17
16.	Table S2	Time constants, preexponential factors and contributions to the signal obtained from emission decays of coated and uncoated InP QDs with surface ligand oleic acid	S-18
17.	Table S3	Time constants, preexponential factors and contributions to the signal obtained from emission decays of coated and uncoated InP QDs with surface ligand oleyl amine	S-19
18.	Table S4	Fitting parameters obtained from multiexponential fits of the femtosecond transient decays at 920 probed wavelength	S-20
19.	Table S5	Fitting parameters obtained from multiexponential fits of the femtosecond transient decays at 480 & 580 nm probed wavelengths	S-20

20.	Table S6	Rate constants, of the different processes, obtained from fitting the transient decays at 920, 480 and 580 nm, upon excitation at 340 nm	S-21
21.	Table S7	Fitting parameters of the nanosecond transient decays at 425 nm	S-21
22.	References	References for the Supporting Information	S-22

S1. Experimental Section

1.1. Materials

Indium acetate ($In(OAc)_3$) (99.99%), Zinc acetate ($Zn(OAc)_3$) (99.99%), 1-octadecene (ODE, >90%) tris(dimethylamino)phosphine (TDMAP, 97%), 1-dodecanethiol (DDT, 98%), oleic acid (OAC, 90%) oleylamine (OAM, 80–90%), have been purchased from Sigma Aldrich (Merck) and used as received without further purification.

1.2. Experimental Section

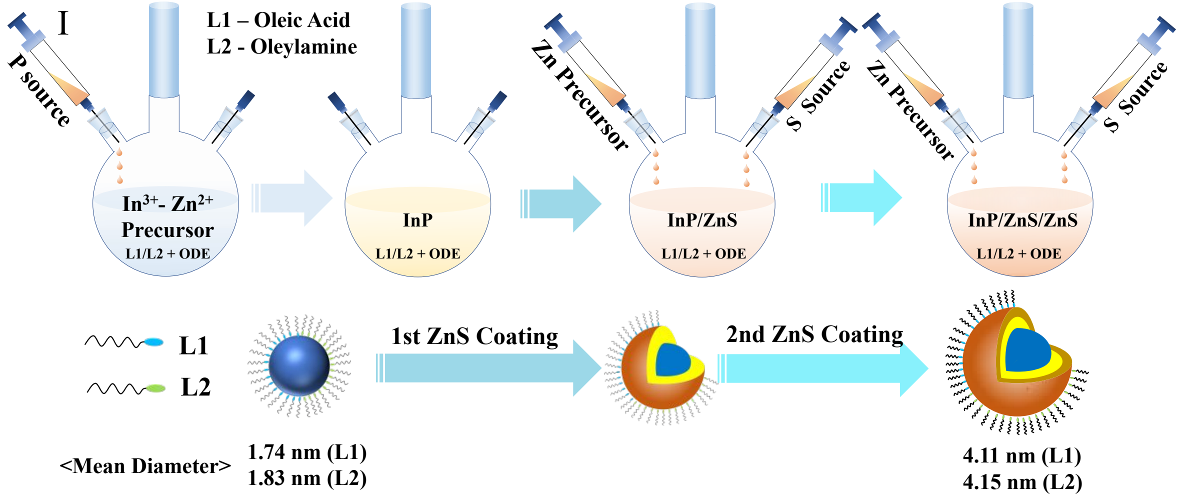

Scheme S1. Top: schematic representation of the synthesis processes of InP core, InP/ZnS, and InP/ZnS/ZnS QDs. Bottom: illustration of uncoated InP, and single-coated InP/ZnS and double-coated InP/ZnS/ZnS QDs.

1.2.1. Synthesis of blue emitting OAC- and OAM-capped InP QDs

In a typical synthesis procedure, 29.2 mg (5 mM) $In(OAc)_3$ and 1.8 mg (0.25 mM) $Zn(OAc)_3$ were mixed in 15 mL of ODE and 0.1 M of OAC (Scheme S1). The mixture was stirred and degassed at 130 °C for half an hour before purging N_2 gas to dissolve completely $In(OAc)_3$ and $Zn(OAc)_3$. After ensuring that the system is homogeneous and in inert atmosphere, the temperature of the solution is increased to 170 °C. Then 0.25 mL of the phosphorous precursor TDMAP was added, and the reaction was then held for 30 minutes to ensure the successful

synthesis of InP QDs. Addition of very small amount of Zn-acetate passivate the surface dangling bonds by forming a In(Zn)P outer layer that protects the InP core from oxidation even after the washing and redispersion process.[1,2,3,4] The formation of this In(Zn)P layer also reduces the probability of secondary nucleation of the InP cores and obstruct Ostawld ripening.[2,4] The InP QDs were then precipitated with ethanol and redispersed in hexane for further studies.

We followed the same procedure for the synthesis of oleylamine (OAM) capped InP QDs but replacing the OAC with 0.1 M of OAM.

1.2.2. Synthesis of blue emitting OAC- and OAM-capped InP/ZnS QDs

The synthesized InP QDs were used to produce OAC capped InP/ZnS QDs. To do so, we mixed a 10 mL solution consisting of $Zn(OAc)_2$ in a mixture of OAC in ODE along with 10 mL of OAC capped InP QDs. The solution was heated at 200 °C for 30 minutes under N_2 environment before adding 1-dodecanthiol (1-DDT). After adding 1-DDT, the temperature of the mixture was quickly increased to 280 °C and kept for 1 hour to coat the first ZnS shell over the InP QDs. The synthesized InP/ZnS QDs were then precipitated in ethanol and redispersed in hexane for further studies.

For the synthesis of OAM-capped InP/ZnS QDs, we have followed the same procedure described above. The only difference is that here we have used a 10 mL solution consisting of $Zn(OAc)_2$ in a mixture of OAM in ODE along with 10 mL of OAM capped InP QDs before adding 1-DDT.

1.2.3. Synthesis of blue emitting OAC- and OAM-capped InP/ZnS/ZnS QDs

The InP/ZnS QDs synthesized in the first step were then employed to produce OAC capped InP/ZnS/ZnS QDs. We mixed a 5 mL solution consisting of $Zn(OAc)_2$ in a mixture of OAC in ODE along with 5 mL of OAC capped InP/ZnS QDs. The solution was heated at 200 °C for 45 minutes under N_2 atmosphere before adding 1-DDT. After the addition of 1-DDT the temperature was increased to 280 °C and kept for 1 hour to ensure the coating of the second ZnS shell over the InP QDs. The synthesized InP/ZnS/ZnS QDs were precipitated in ethanol and redispersed in hexane.

Blue emitting OAM-capped InP/ZnS/ZnS QDs were synthesized using the same procedure. The only difference is the replacement of OAC by OAM.

1.3. Methods

1.3.1. Transmission Electron Microscopy (TEM)

High-resolution TEM (HRTEM) images of the samples were recorded using a JEOL 2100 microscope (JEOL, Tokyo, JP) operating at 200 kV and equipped with an Orius Gatan 2x2 MPi

digital camera (Gatan, Pleasanton, US-CA). Specimens were prepared by depositing a drop of sample suspensions onto holey carbon Cu-grids (SPI, 200 mesh). HRTEM images were collected under low-dose conditions to minimize beam damage. The average sizes of the QDs with the two different surface ligands have been averaged after measuring at least 150 QDs.

1.3.2. Steady-state absorption, emission and picosecond (ps) time resolved observation

A JASCO V-670 spectrophotometer was used to record steady-state UV-visible absorption spectra using 10-mm path length quartz cuvettes. The excitation and emission spectra were recorded on a Fluoromax-4 (Jobin-Yvone) spectrofluorometer using a quartz cuvette of 10-mm path length. Excited-state lifetimes were measured using a ps time-correlated single-photon counting system (FluoTime 200, PicoQuant) described previously.[5] The samples were excited by a 0.8 mW, 40 ps pulsed (20 MHz) LDH 400 laser centered at 371 nm. The fluorescence signal was collected at magic angle and at different observation wavelengths. The instrument response function (IRF) was 70 ps. The decays were deconvoluted and fitted to a multiexponential function using the FLUOFIT package (PicoQuant), which allows single and global fits. The quality of the fits as well as the number of exponentials were checked based on the reduced χ^2 values (which were always below <1.2) and the distributions of the residuals.

1.3.3. Fluorescence Upconversion Spectroscopy

The femtosecond (fs) time-resolved emission transients were collected using a fluorescence up-conversion setup (FOG100, CDP Systems) described elsewhere.[6,7] The samples were excited at 360 nm by a fs-pulse from the second harmonic of Ti:Sapphire oscillator (MaiTai Spectra Physics) output (720 nm). The samples were placed in a 1-mm rotating cell to avoid re-excitation and photodegradation, The IRF of the setup is ∼180 fs measured as the Raman signal of the solvent. The recorded transients at selected gated wavelengths were analyzed by convoluting a multiexponential function with the IRF to fit the experimental data. The estimated errors for the calculated time constants were below 15% in all cases.

1.3.4. Femtosecond Transient Absorption Spectroscopy

The used fs transient UV−vis−NIR absorption setup has been described elsewhere.[8] Briefly, it consists of a Ti:Sapphire oscillator (TISSA 50, CDP Systems) pumped by a 5 W diode laser (Verdi 5, Coherent). The seed pulse (30 fs, 450 mW at 86 MHz) centered at 800 nm is directed to chirped pulse amplification system (Legend-USP, Coherent). The amplified fundamental beam (50 fs, ~3 W at 1 kHz) is then split by a beam splitter and the main portion (2.7 W) is directed through an optical parametric amplifier for wavelength conversion (TOPAS, Light

Conversion). A small portion of the rest of the fundamental beam (~200 μW) was directed to Sapphire crystal for white light continuum generation. The used pump intensity was ∼500 μW (spot size at the sample was 280 μm) and the excitation wavelength was 340 nm. The instrumental response function (IRF) was 160 fs. All spectra analyzed in the UV−vis region were corrected for the chirp of the white light continuum. Transient absorption measurements were performed in the spectral ranges of 430−620 (UV−vis region) and 870 – 1050 nm (NIR). To avoid sample degradation, the samples were placed in a 1-mm rotating cell. The data were analyzed using a multiexponential global fit program. The quality of the global fit was checked by examining the fits at different wavelengths and χ^2.

1.3.5. Flash Photolysis Measurements

The nanosecond to second flash photolysis setup was described elsewhere.[9] Laser flash photolysis experiment was carried out in a LKS.60 laser flash photolysis spectrometer (Applied Photophysics) coupled with a Vibrant (HE) 355 II laser (Opotek) as pump pulse source. The third harmonic (355 nm) of the a Q-switched Nd:YAG laser (Brilliant, Quantel) output was used as excitation. The probing light source is a 150 W xenon arc lamp. The light transmitted through the sample is separated by a monochromator and detected by visible photomultipliers (Applied Photophysics R928) attached to a digital oscilloscope (Agilent Infiniium DS08064A, 600 MHz, 4 GSa/s). The measured IRF of the system is ~ 8 ns.

SN1:

The average radiative (k_r) and non-radiative (k_{nr}) rate constants were calculated using the QY and the averaged lifetime, τ_{avg}, using the following two equations:[10,11]

$$\frac{1}{\tau_r} = k_r = \frac{\Phi}{\tau_{avg}} \qquad \text{(Eq. 1)}$$

$$k_{nr} = \frac{1}{\tau_{nr}} = \frac{1}{\tau_{avg}} - \frac{1}{\tau_r} \qquad \text{(Eq. 2)}$$

SN2:

The average number of photons absorbed per quantum dot per pulse is determined by the following procedure.[12,13] The data presented Figure 4, 5 and 6 in the main article were measured at a pump power, P, of 500 μW with a repetition rate, R, of 3 kHz of the laser fundamental, where every second pump pulse was blocked by the chopper. The energy per pulse (E_{pulse}) is thus given by,

$$E_{pulse} = \frac{P}{R} = \frac{0.500\, mW}{1.5 \times 10^3\, s^{-1}} = 333\, nJ \qquad \text{(Eq. 3)}$$

The pump area was determined from the beam diameter measured by the knife-edge technique ((d)=300 μm). The area of the pump (A_{pump}) pulse at the sample is thus given by,

$$A_{pump} = \pi\left({}^{d}/{}_{2}\right)^2 = 3.14 \times \left({}^{0.030}/{}_{2}\right)^2 cm^2 = 7.01 \times 10^{-4}\, cm^2 \quad \text{(Eq. 4)}$$

The pump fluence, $F_{E/A}$ (average energy per pulse per area), is given by the ratio between the pump pulse energy and the pump area at the sample. Note that we are not considering the peak power and pulse duration,

$$F_{E/_A} = \frac{E_{pulse}}{A_{pump}} = \frac{333\, nJ}{7.01 \times 10^{-4} cm^2} = 475\, \mu J\, cm^{-2} \quad \text{(Eq. 5)}$$

The energy per photon, E_{phot}, at a wavelength of 400 nm is given by the Planck-Einstein relation,

$$E_{Phot} = \frac{hc}{\lambda} = \frac{(6.6 \times 10^{-34} J\, s)(3 \times 10^{8}\, m\, s^{-1})}{340 \times 10^{-9}\, m} = 5.88 \times 10^{-19} J \quad \text{(Eq. 6)}$$

The photon fluence (per pulse per area) is thus given by the ratio between the pump fluence and the photon energy, according to,

$$F_{fluence} = \frac{F_{E/_A}}{E_{Phot}} = \frac{475\, \mu J\, cm^{-2}}{5.88 \times 10^{-19} J} = 8.1 \times 10^{14}\, cm^{-2} \quad \text{(Eq. 7)}$$

The absorption cross section (σ_{abs}) of the QDs has been determind by calculating the concentration of the QDs with the values of the volume of the unit cell and volume of an individual QD.[13,14] The concentration of the QDs is found to be 1.95×10^{-6}M. The value of σ_{abs} (cm^2) was obtained from the equation $\sigma_{abs} = \frac{2303\, \varepsilon_\lambda}{N_A}$ and, the valuev of σ_{abs}, at 340 nm is found to be □0.98×10^{-15} cm^2.

The average number of photons absorbed per QD per pulse, $\langle N \rangle$, was then estimated by using the following equation,

$$\langle N \rangle = F_{fluence} \times \sigma_{abs} = (8.1 \times 10^{14}\, cm^{-2}) \times (0.98 \times 10^{-15} cm^2) \cong 0.8 \quad \text{(Eq. 8)}$$

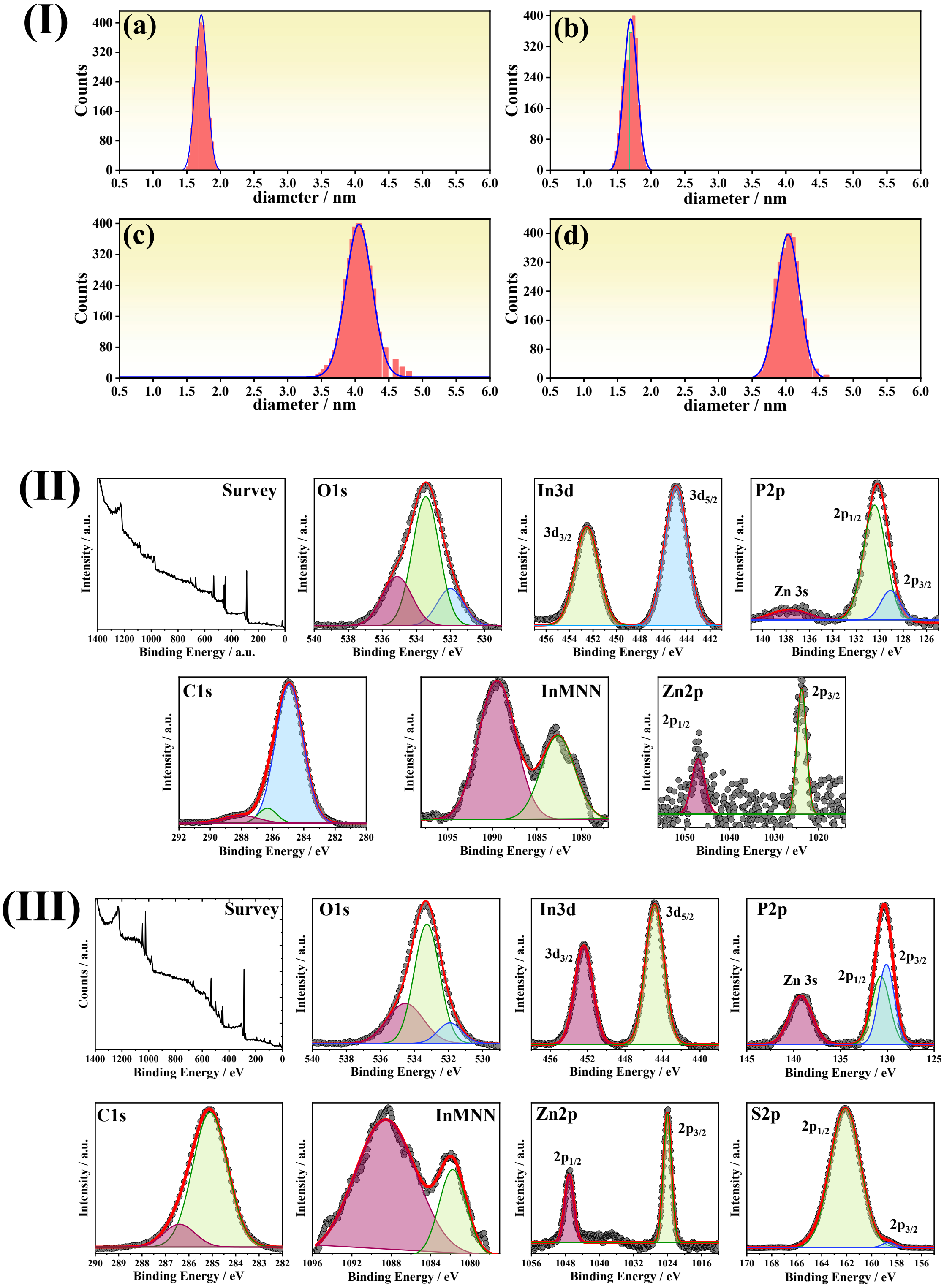

Figure S1. (I) Size distribution histogram of InP core and InP/ZnS/ZnS QDs with surface ligands OAC (a,b) and OAM (d,e), respectively. (II) Full XPS spectrum of the ultrasmall InP QDs and (III) InP/ZnS/ZnS QDs and high-resolution scan of the present elements. In the

deconvoluted spectra, the blue colour represents the lowest binding energy whereas green and red ones the highest energies.

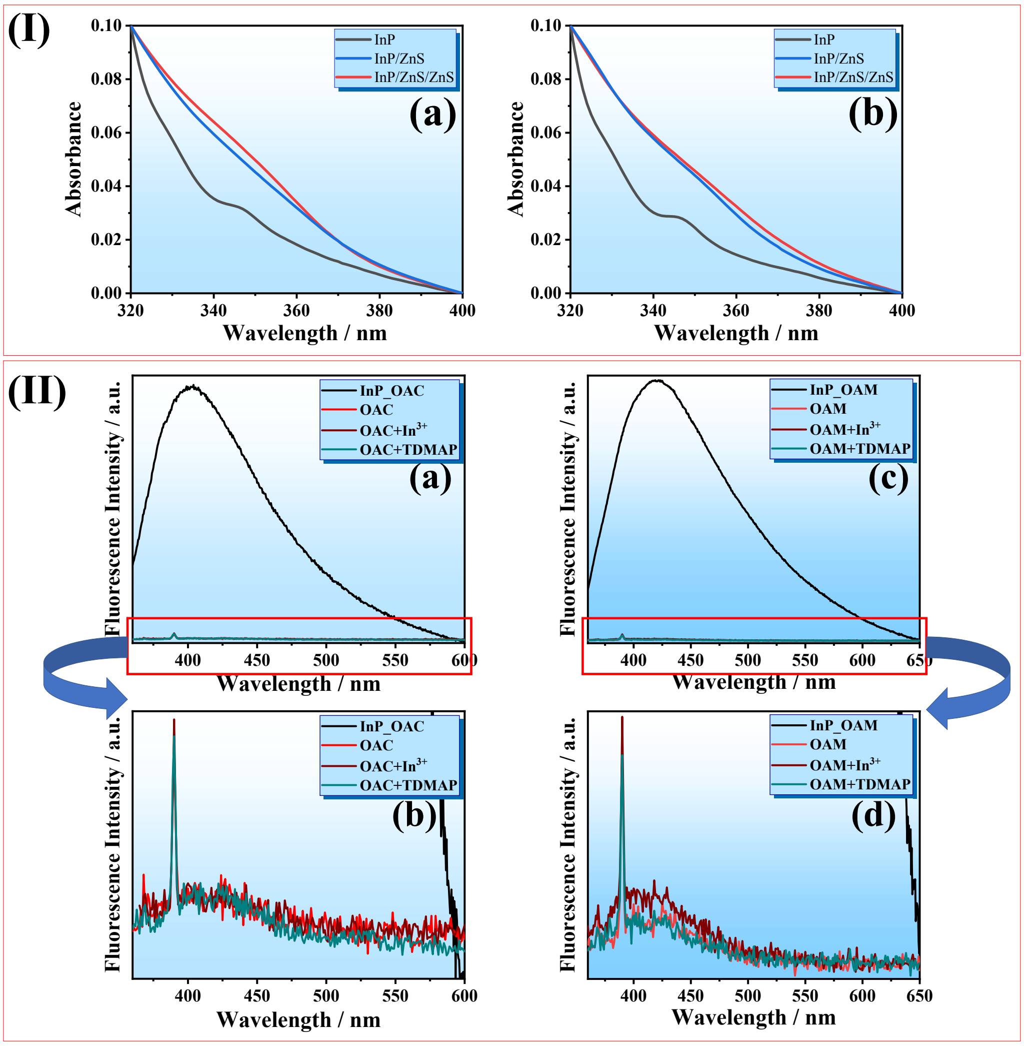

Figure S2. (I) Change in the absorption spectra of InP QDs upon successive surface passivations by ZnS shell in the presence of ligand OAC (a) and OAM (b). (II) Demonstration of the lack of emission of the used chemical components when used separately: Emission spectra of (a) OAC, In^{3+} and TDMAP in the presence of OAC & InP with surface ligand OAC; (b) OAM, In^{3+} and TDMAP in the presence of OAM & InP with surface ligand OAM; (c) and (d) are the zoomed in part of the non-emissive components.

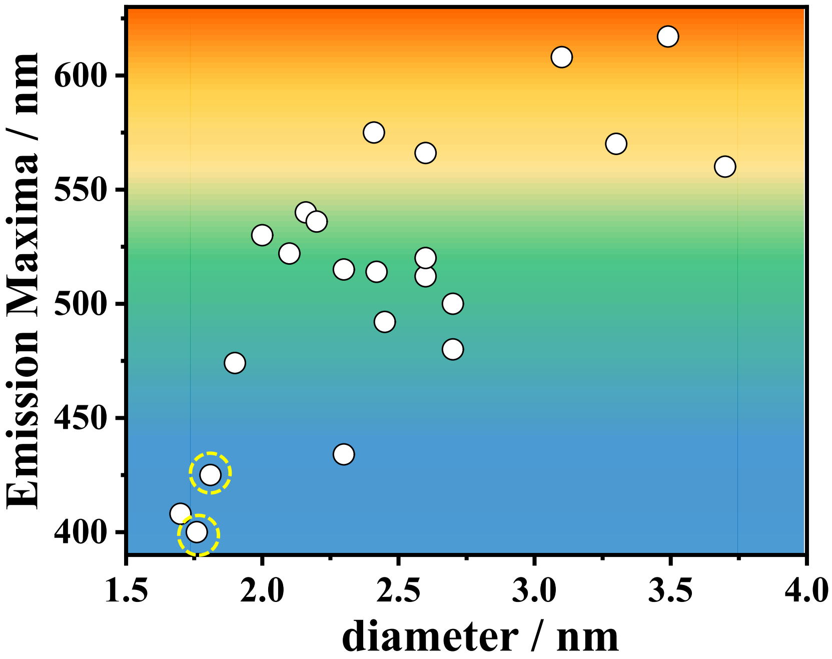

Figure S3. Graphical illustration of the emission color of InP core QDs with respect to their sizes. The data are from references in Table S1. The circled dots are from this work.

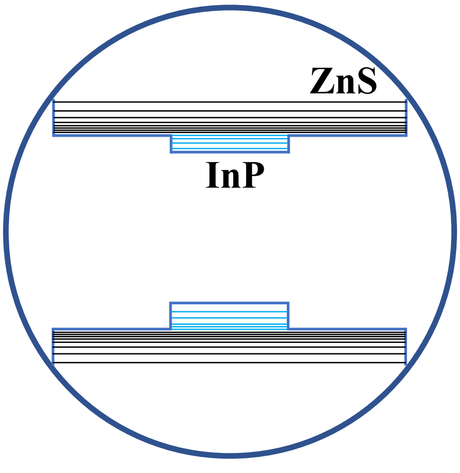

Scheme S2. Schmatic representation of the energy bandgap in InP/ZnS/ZnS QDs.

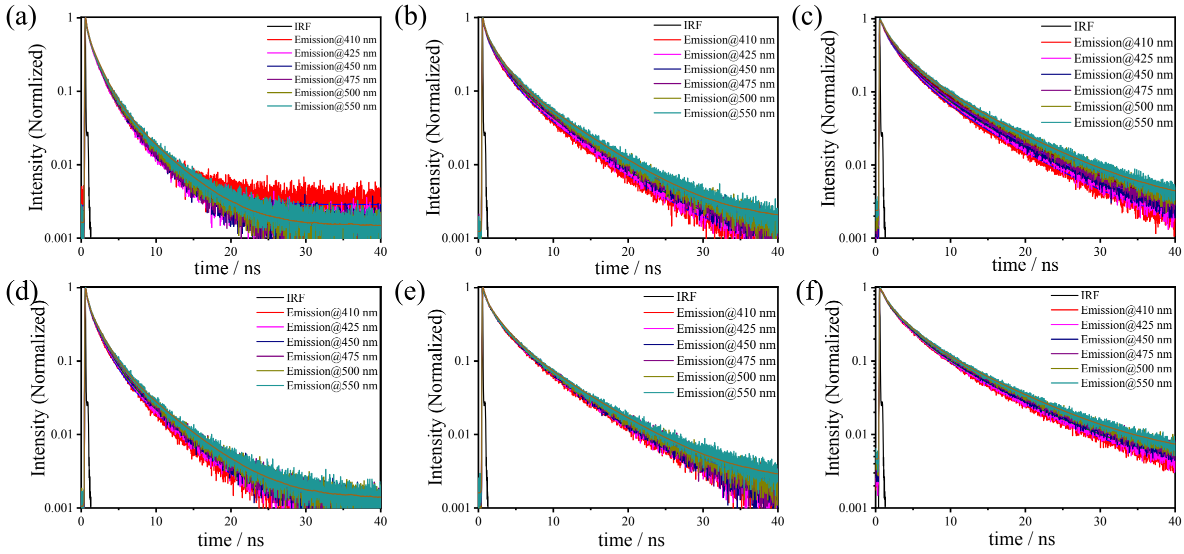

Figure S4. Magic-angle time-resolved photoluminescence (PL) decays of InP, InP/ZnS and InP/ZnS/ZnS QDs with (a,b,c) OAM and (c,d,e) OAC as surface ligands, monitored at different wavelengths (410-550 nm) and upon excitation at 371 nm. The IRF is the instrument response function (70 ps). The solid lines are from the best global fit using a multiexponential function.

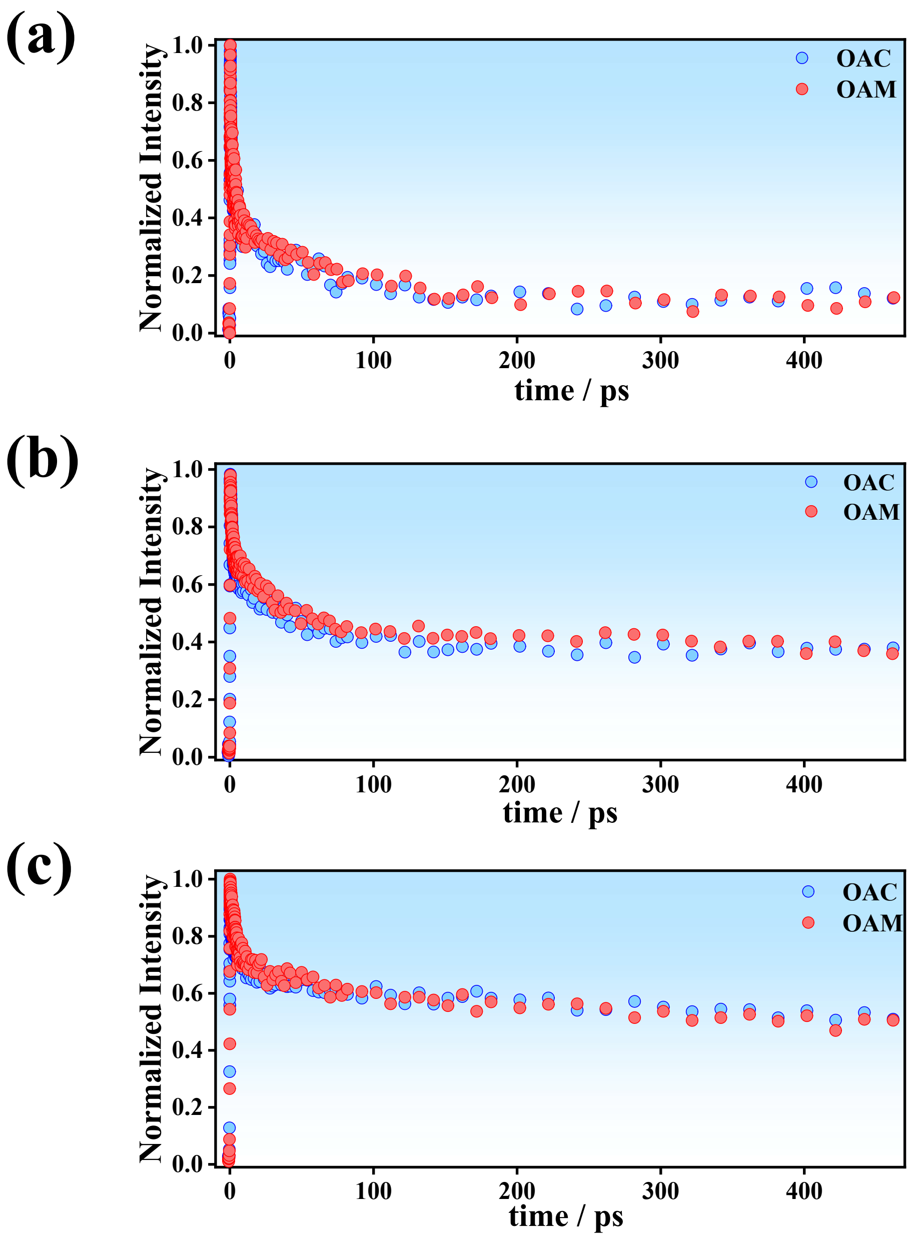

Figure S5. Femtosecond emission transients of InP (a), InP/ZnS (b) and InP/ZnS/ZnS (c) QDs with surface ligands OAC and OAM upon excitation at 360 nm. The monitored emission wavelength was 425 nm.

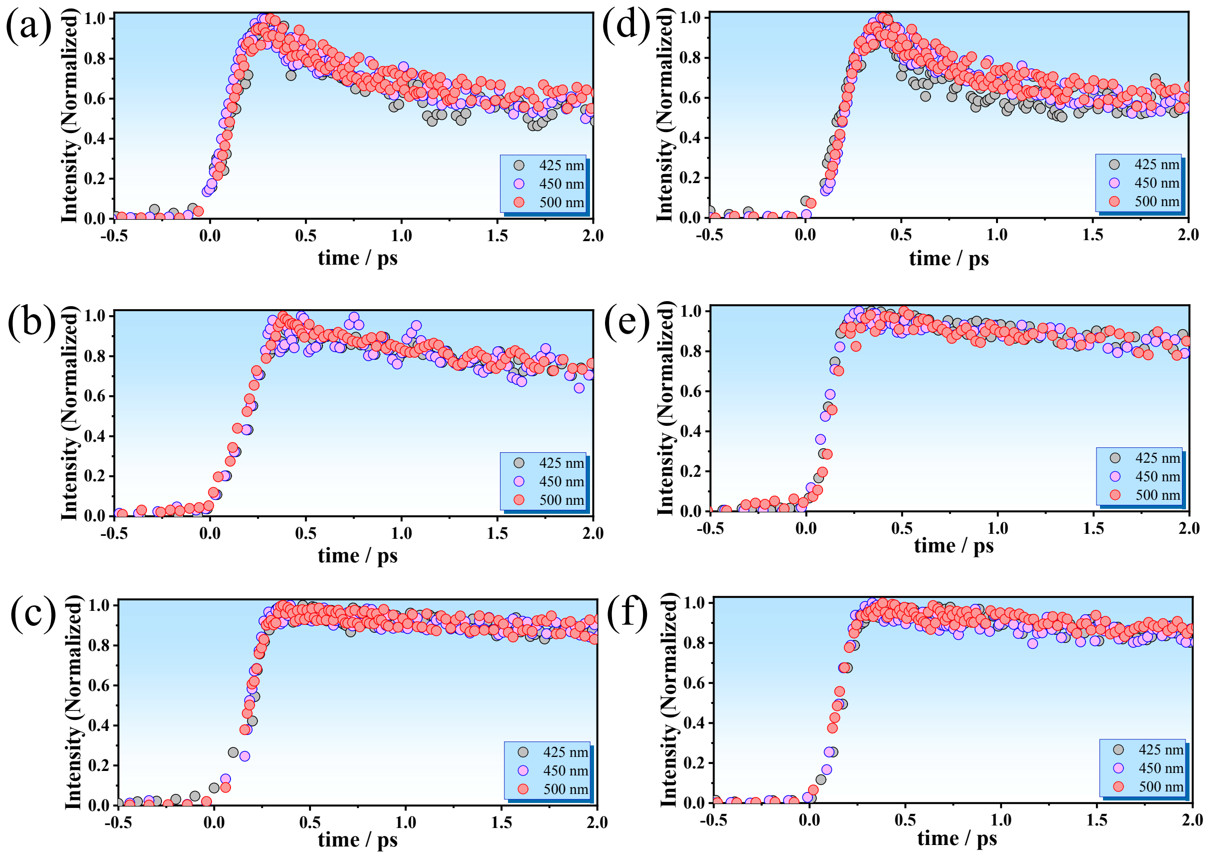

Figure S6. Short window femtosecond emission transients of InP, InP/ZnS, and InP/ZnS/ZnS QDs at different emission wavelengths (425, 450 and 500 nm) with surface ligands OAC (a,b,c) and OAM (d,e,f) upon excitation at 360 nm.

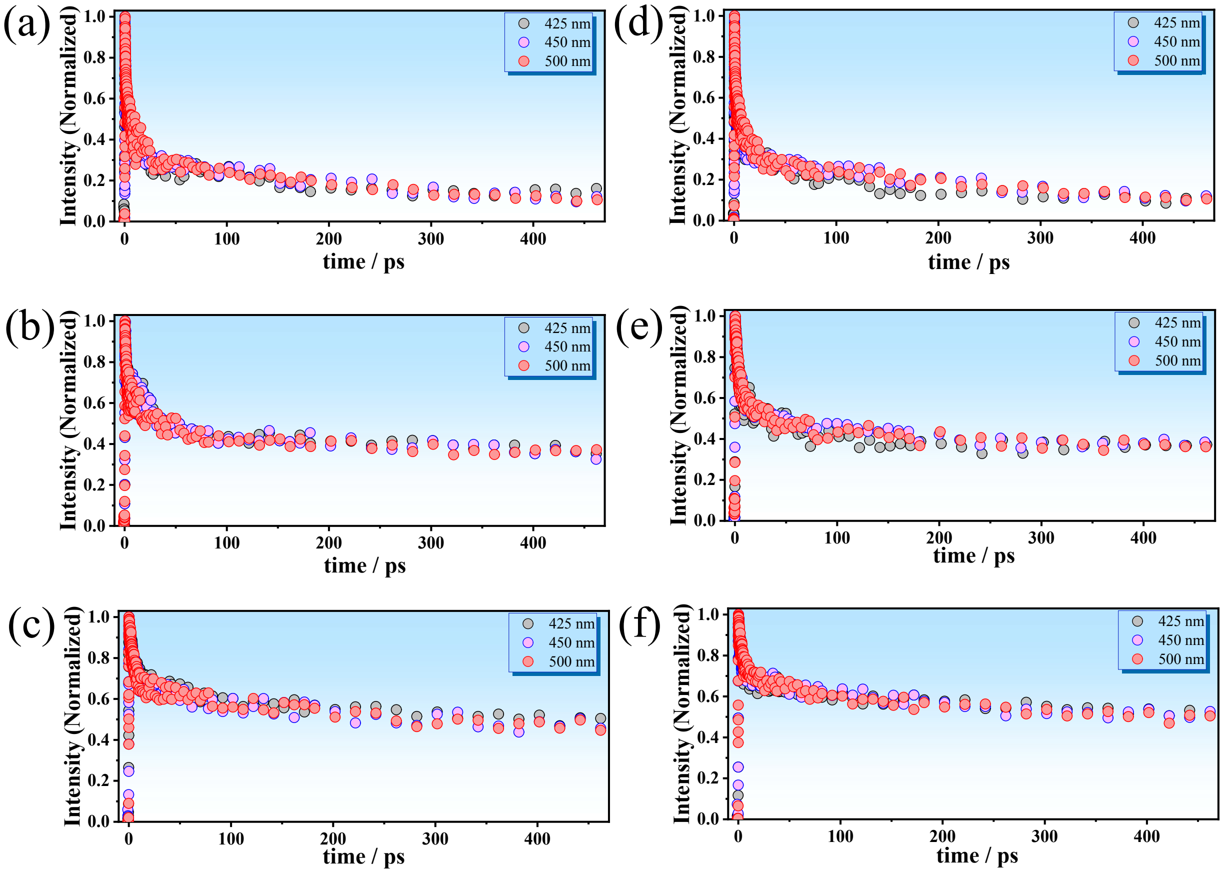

Figure S7. Femtosecond emission transients of InP, InP/ZnS and InP/ZnS/ZnS QDs at different emission wavelengths (425, 450 and 500 nm) with surface ligands OAC (a,b,c) and OAM (d,e,f) upon excitation at 360 nm.

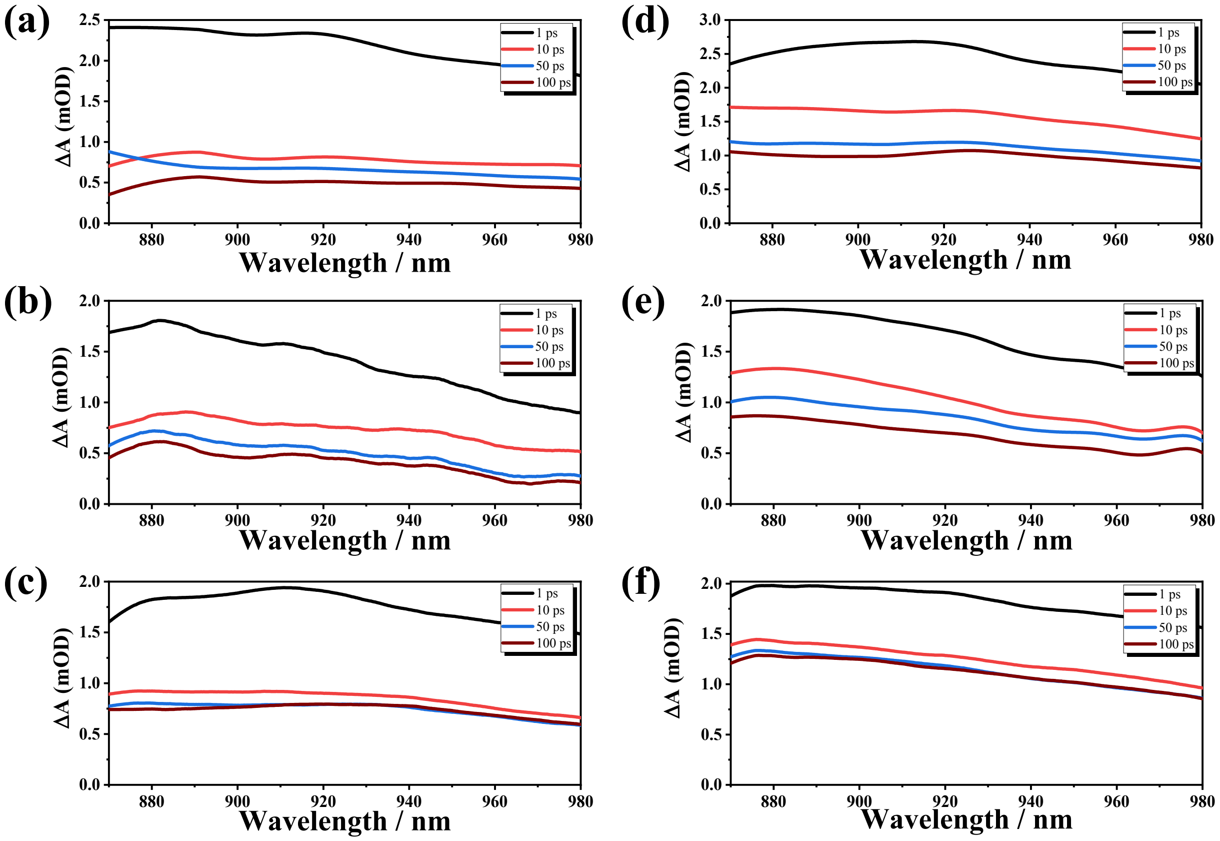

Figure S8. (I) Femtosecond transient absorption spectra of the InP QDs, (a) uncoated, (b) coated with one and (c) with two ZnS shells upon 340 nm excitation with the surface ligand OAC probed in the 870-980 nm region. (II) Transient absorption spectra of the InP QDs, (a) uncoated, (b) coated with one and (c) with two ZnS shells upon 340 nm excitation with the surface ligand OAM probed in the 870-980 nm region.

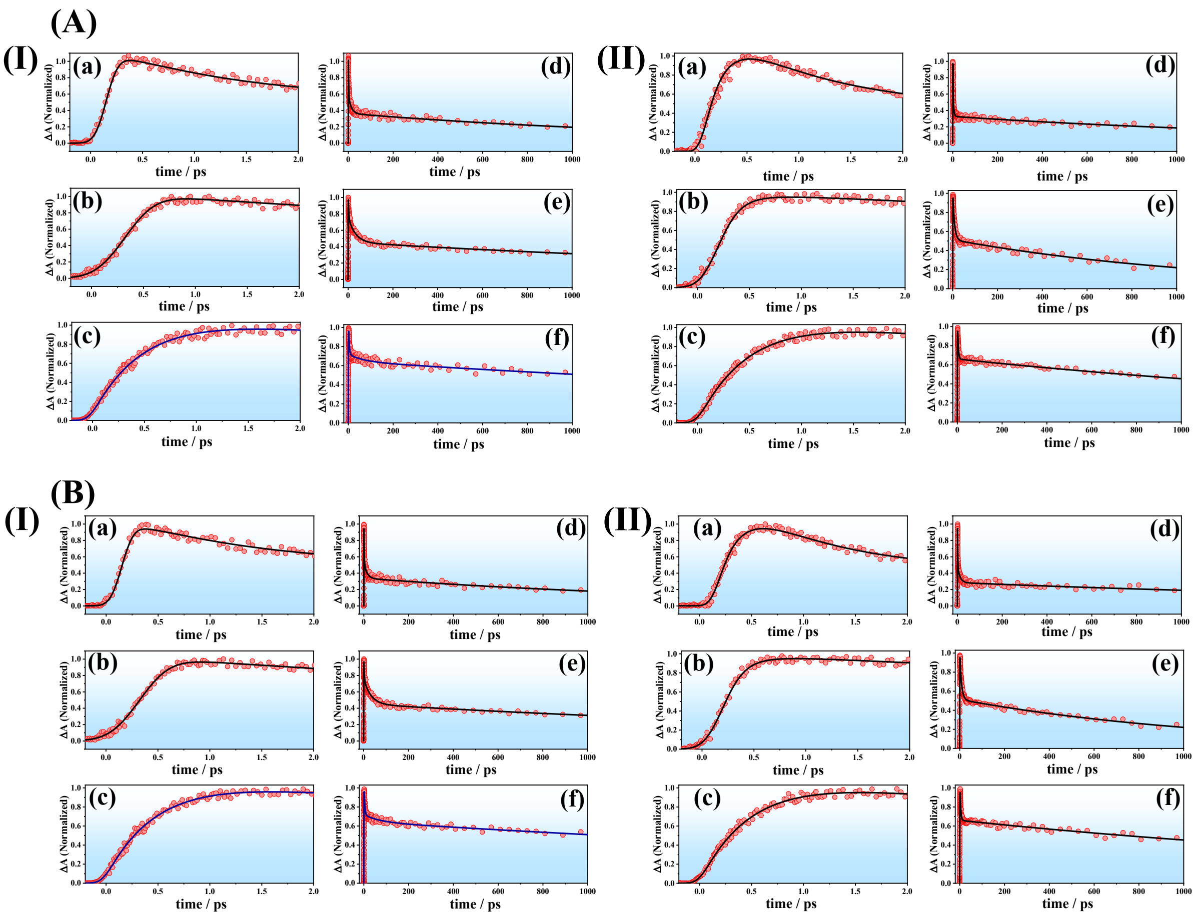

Figure S9. (A) (I) & (II) Early time TA dynamics probed at 870 nm of (a) InP, (b) InP/ZnS, and (c) InP/ZnS/ZnS QDs using OAC and OAM as surface ligands, respectively. (d), (e), (f) Are the full-window data for the same InP, InP/ZnS, InP/ZnS/ZnS QDs, respectively. (II) Early time TA dynamics probed at 870 nm of (a) InP, (b) InP/ZnS, and (c) InP/ZnS/ZnS QDs with OAM as surface ligand. (d), (e), (f) are the full-scale data for the same InP, InP/ZnS, InP/ZnS/ZnS QDs, respectively. (B) (II) Early time TA dynamics probed at 970 nm of (a) InP, (b) InP/ZnS, and (c) InP/ZnS/ZnS QDs using OAC and OAM as surface ligands respectively. (d), (e), (f) are the full-scale data for the same InP, InP/ZnS, InP/ZnS/ZnS QDs, respectively. (II) Early time TA dynamics probed at 970 nm of (a) InP, (b) InP/ZnS and (c) InP/ZnS/ZnS QDs with OAM as surface ligand. (d), (e), (f) are the full-scale data for the same InP, InP/ZnS, InP/ZnS/ZnS QDs, respectively. All the experiments were carried out upon excitation at 340 nm. The solid lines are from the best fits using a multiexponential function.

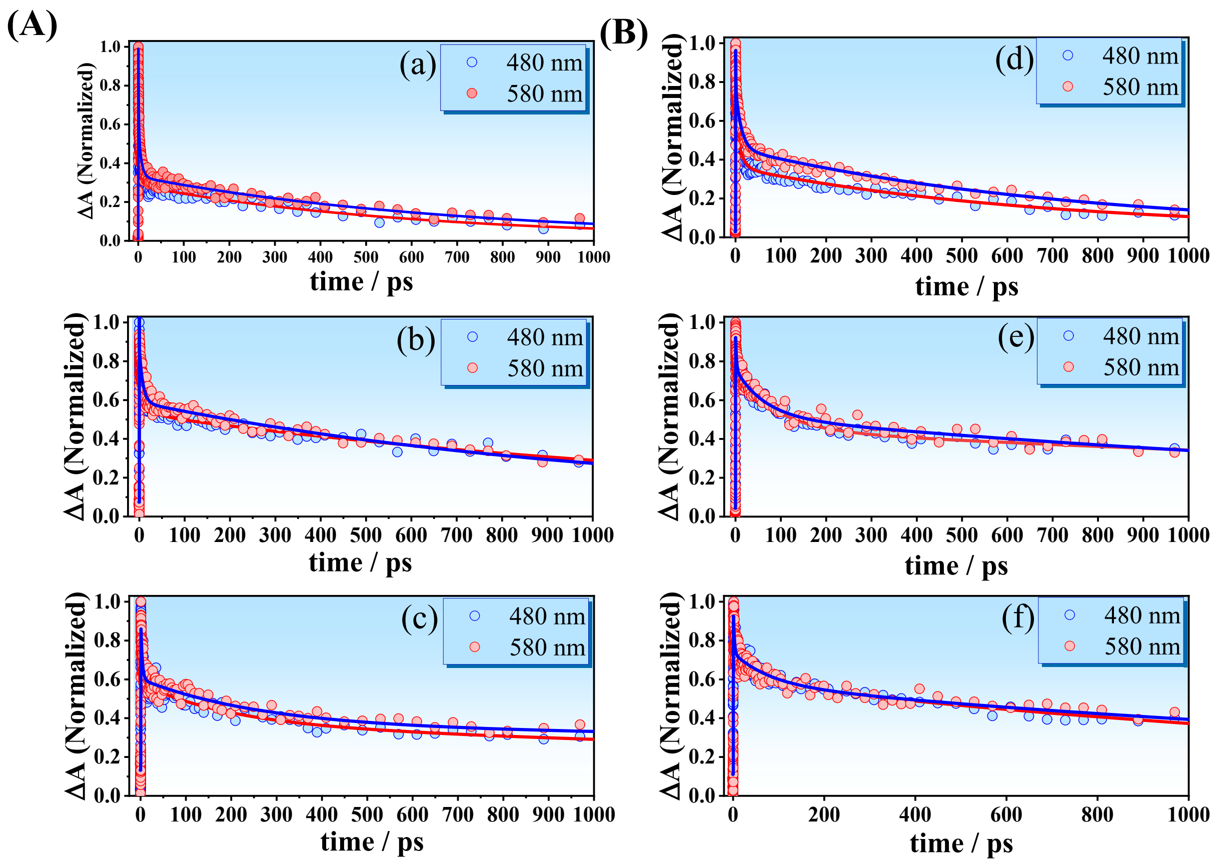

Figure S10. (A) Femtosecond transient decays of (a) InP, (b) InP/ZnS, and (c) InP/ZnS/ZnS QDs with surface ligand OAC. (B) Femtosecond transient decays of (d) InP, (e) InP/ZnS, and (f) InP/ZnS/ZnS QDs with surface ligand OAM. The probed wavelengths were 480 and 580 nm. The solid lines are from the best multiexponential fits.

Table S1. Values of size and emission intensity maximum wavelength of the synthesized InP QDs in this study and reported elsewhere, where TDMAP has been used as the source of phosphorus.

**Size (nm)**	**Emission Wavelength Maximum (nm)**	**Reference**
3.7	560	J. Mater. Chem. C, **2021**, *9*, 9599–9609
3.49	617	Small **2022**, *18*, 2108120
3.3	570	Nature, **2019**, *575*, 634–638
3.1	608	J. Mater. Chem. C, **2022**, *10*, 2213–2222
2.7	500	Nature, **2019**, *575*, 634–638
2.7	480	J. Phys. Chem. Lett. **2020**, *11*, 960–967
2.6	512	Small **2022**, *18*, 2108120
2.6	520	ACS Nano **2018**, *12*, 8104−8114
2.6	566	J. Mater. Chem. C, **2022**, *10*, 2213–2222
2.45	492	Small **2022**, *18*, 2108120
2.42	514	Nanoscale, **2022**, *14*, 9900–9909
2.41	575	J. Phys. Chem. Lett. **2021**, *12*, 7194−7200
2.3	434	Adv. Funct. Mater. **2021**, *31*, 2008453
2.3	515	Small **2022**, *18*, 2105492
2.2	536	J. Mater. Chem. C, **2022**, *10*, 2213–2222
2.16	540	Adv. Sci. **2022**, *9*, 2200959
2.1	522	ACS Appl. Mater. Interfaces **2022**, *14*, 12479−12487
2	530	ACS Appl. Nano Mater. **2020**, *3*, 1972−1980
1.9	474	Adv. Optical Mater. **2022**, 2200685
1.7 (InP Nanocluster)	408	Nature Comm., **2020**, *11*, 3127
1.76	400	This work

Table S2. Values of time constants (τ_i), normalized (to 1) pre-exponential factors (a_i), and contributions ($c_i = \tau_i \times a_i/\sum(\tau_i \times a_i)$) to the signal obtained from a global multiexponential fit for the InP, InP/ZnS and InP/ZnS/ZnS QDs with surface ligand oleic acid (OAC) upon excitation at 371 nm. The observation wavelengths are indicated. The estimated errors in the lifetimes are around 10-15%.

**Ligand**	**QD**	**Wavelength (nm)**	**τ_1 (ns)**	**a_1**	**c_1**	**τ_2 (ns)**	**a_2**	**c_2**	**τ_3 (ns)**	**a_3**	**c_3**	**χ^2**
Oleic Acid	InP	410	0.17	0.53	0.10	1.31	0.39	0.56	4.08	0.08	0.35	1.12
		425	0.17	0.54	0.10	1.31	0.38	0.54	4.08	0.08	0.37	1.13
		450	0.17	0.52	0.09	1.31	0.39	0.53	4.08	0.09	0.38	1.11
		475	0.17	0.52	0.09	1.31	0.38	0.52	4.08	0.09	0.39	1.12
		500	0.17	0.52	0.09	1.31	0.38	0.49	4.08	0.10	0.42	1.12
		550	0.17	0.52	0.08	1.31	0.36	0.47	4.08	0.11	0.45	1.13
	InP/ZnS	410	0.24	0.36	0.05	1.97	0.48	0.47	5.71	0.16	0.48	1.14
		425	0.24	0.36	0.05	1.97	0.48	0.48	5.71	0.16	0.48	1.12
		450	0.24	0.36	0.05	1.97	0.47	0.46	5.71	0.17	0.49	1.12
		475	0.24	0.36	0.05	1.97	0.46	0.45	5.71	0.18	0.50	1.10
		500	0.24	0.35	0.04	1.97	0.45	0.44	5.71	0.20	0.52	1.13
		550	0.24	0.34	0.04	1.97	0.43	0.42	5.71	0.22	0.54	1.12
	InP/ZnS/ZnS	410	0.43	0.38	0.06	2.46	0.47	0.47	7.68	0.15	0.47	1.10
		425	0.43	0.36	0.06	2.46	0.47	0.44	7.68	0.17	0.50	1.12
		450	0.43	0.35	0.05	2.46	0.46	0.42	7.68	0.19	0.53	1.12
		475	0.43	0.33	0.05	2.46	0.46	0.40	7.68	0.21	0.55	1.12
		500	0.43	0.32	0.04	2.46	0.45	0.37	7.68	0.23	0.58	1.10
		550	0.43	0.31	0.04	2.46	0.44	0.35	7.68	0.24	0.60	1.10

Table S3. Values of time constants (τ_i), normalized (to 1) pre-exponential factors (a_i), and contributions ($c_i = \tau_i \times a_i / \sum(\tau_i \times a_i)$) to the signal obtained from a global multiexponential fit for the InP, InP/ZnS and InP/ZnS/ZnS QDs with surface ligand oleyl amine (OAM) upon excitation at 371 nm. The observation wavelengths are indicated. The estimated errors in the lifetimes are around 10-15%.

Ligand	QD	Wavelength (nm)	τ_1 (ns)	a_1	c_1	τ_2 (ns)	a_2	c_2	τ_3 (ns)	a_3	c_3	χ^2
Oleyl Amine	InP	410	0.21	0.51	0.12	1.52	0.38	0.54	4.92	0.08	0.34	1.12
		425	0.21	0.51	0.12	1.52	0.38	0.54	4.92	0.08	0.34	1.13
		450	0.21	0.51	0.11	1.52	0.38	0.53	4.92	0.09	0.36	1.11
		475	0.21	0.50	0.10	1.52	0.37	0.52	4.92	0.10	0.38	1.12
		500	0.21	0.50	0.09	1.52	0.37	0.50	4.92	0.10	0.41	1.12
		550	0.21	0.49	0.08	1.52	0.36	0.48	4.92	0.11	0.44	1.13
	InP/ZnS	410	0.30	0.38	0.07	2.05	0.46	0.47	6.63	0.16	0.46	1.14
		425	0.30	0.38	0.07	2.05	0.48	0.47	6.63	0.16	0.46	1.12
		450	0.30	0.37	0.07	2.05	0.47	0.46	6.63	0.17	0.47	1.12
		475	0.30	0.36	0.06	2.05	0.46	0.45	6.63	0.18	0.49	1.10
		500	0.30	0.36	0.06	2.05	0.45	0.44	6.63	0.20	0.50	1.13
		550	0.30	0.35	0.05	2.05	0.43	0.42	6.63	0.22	0.53	1.12
	InP/ZnS/ZnS	410	0.60	0.30	0.05	2.95	0.47	0.46	8.71	0.15	0.49	1.10
		425	0.60	0.30	0.05	2.95	0.47	0.44	8.71	0.17	0.51	1.12
		450	0.60	0.29	0.05	2.95	0.46	0.42	8.71	0.19	0.53	1.12
		475	0.60	0.29	0.04	2.95	0.46	0.40	8.71	0.21	0.56	1.12
		500	0.60	0.29	0.04	2.95	0.45	0.37	8.71	0.23	0.59	1.10
		550	0.60	0.28	0.04	2.95	0.44	0.34	8.71	0.24	0.62	1.10

Table S4. Values of the time constants and pre-exponential factors (a_i) obtained from the best fit of the transients of InP, InP/ZnS, and InP/ZnS/ZnS QDs upon excitation at 340 nm and observation at 920 nm. The estimated errors in the lifetimes are around 10-15%.

Ligand	QD	τ_1 (ps)	a_1	τ_2 (ps)	a_2	τ_3 (ns)	a_3
Oleic Acid	InP	0.20	-1	2	0.72	> 1	0.28
	InP/ZnS	0.40	-1	5	0.60	> 1	0.40
	InP/ZnS/ZnS	0.70	-1	8	0.40	> 1	0.60
Oleyl Amine	InP	0.20	-1	2	0.70	> 1	0.30
	InP/ZnS	0.40	-1	5	0.55	> 1	0.45
	InP/ZnS/ZnS	0.70	-1	8	0.40	> 1	0.60

Table S5. Values of the time constants and normalized (to 1) pre-exponential factors (a_i) obtained from the best fit of the transients of InP, InP/ZnS, and InP/ZnS/ZnS QDs upon excitation at 340 nm and observation at 480 and 580 nm. The estimated errors in the lifetimes are around 10-15%.

Ligand	QD	Wavelength (nm)	τ_1 (ps)	a_1	τ_2 (ps)	a_2	τ_3 (ns)	a_3	τ_4 (ps)	a_4
Oleic Acid	InP	480	1.00	0.40	12	0.50	0.60	0.10		
		580	1.50	0.38	15	0.52	0.80	0.10		
	InP/ZnS	480	0.30	0.20	30	0.50	> 1	0.30		
		580	2.00	0.30	35	0.30	> 1	0.4	0.20	-1
	InP/ZnS/ZnS	480	0.50	0.20	90	0.20	> 1	0.60		
		580	3.50	0.15	140	0.25	> 1	0.60	0.30	-1
Oleyl Amine	InP	480	1.00	0.33	14	0.55	0.60	0.12		
		580	1.50	0.32	18	0.57	0.80	0.11		
	InP/ZnS	480	0.30	0.23	35	0.50	> 1	0.27		
		580	2.00	0.28	42	0.45	> 1	0.27	0.20	-1
	InP/ZnS/ZnS	480	0.50	0.20	120	0.28	> 1	0.52		
		580	4.00	0.25	165	0.25	> 1	0.50	0.30	-1

Table S6. Rates for different processes obtained from fitting the transient decays at 920, 480 and 580 nm, upon excitation at 340 nm. The estimated errors in the rate constants are around 10-15%.

Ligand	QD	k_{HET} (ps^{-1})	k_{CET} (ps^{-1})	k_{CHT} (10^{-1} ps^{-1})	k_{AR} (10^{-2} ps^{-1})
Oleic Acid	InP	50	1.00	1.70	6.67
	InP/ZnS	20	0.50	0.31	2.86
	InP/ZnS/ZnS	12.5	0.29	0.22	0.71
Oleyl Amine	InP	50	1.00	1.20	5.57
	InP/ZnS	20	0.50	0.28	2.38
	InP/ZnS/ZnS	12.5	0.25	0.15	0.61

Table S7. Values of time constants (τ_i) and normalized (to 1) pre-exponential factors (a_i) obtained from a biexponential fit of the flash photolysis transients of InP, InP/ZnS, InP/ZnS/ZnS QDs with surface ligands oleic acid and oleyl amine upon excitation at 355 nm. The observation wavelength was 425 nm. The estimated errors in the lifetimes are around 10-15%.

**Ligand**	**QD**	**Atmosphere**	**τ_1 (μs)**	**a_1**	**τ_2 (μs)**	**a_2**
Oleic Acid	InP	Air	0.14	0.71	1.46	0.29
		N_2	0.26	0.51	1.54	0.49
		O_2	0.02	0.90	1.23	0.10
	InP/ZnS	Air	0.19	0.80	1.50	0.20
		N_2	0.20	0.78	1.51	0.22
		O_2	0.18	0.81	1.50	0.19
	InP/ZnS/ZnS	Air	0.26	0.54	1.60	0.46
		N_2	0.26	0.55	1.60	0.45
		O_2	0.26	0.54	1.60	0.46
Oleyl Amine	InP	Air	0.18	0.72	1.54	0.28
		N_2	0.24	0.32	1.56	0.68
		O_2	0.07	0.84	1.53	0.16
	InP/ZnS	Air	0.30	0.60	2.01	0.40
		N_2	0.30	0.61	2.00	0.39
		O_2	0.30	0.60	2.01	0.40
	InP/ZnS/ZnS	Air	0.32	0.65	2.02	0.35
		N_2	0.33	0.64	2.03	0.36
		O_2	0.32	0.65	2.02	0.35

References:

(1) Kirkwood, N.; De Backer, A.; Altantzis, T.; Winckelmans, N.; Longo, A.; Antolinez, F. V.; Rabouw, F. T.; De Trizio, L.; Geuchies, J. J.; Mulder, J. T.; Renaud, N.; Bals, S.; Manna, L.; Houtepen, A. J. Locating and Controlling the Zn Content in In(Zn)P Quantum Dots. *Chem. Mater.* **2020**, *32*, 557–565.

(2) Choi, Y.; Kim, D.; Shin, Y. S.; Lee, W.; Orr, S.; Kim, J. Y.; Park, J. Highly Luminescent Red-Emitting In(Zn)P Quantum Dots Using Zinc Oxo Cluster: Synthesis and Application to Light-Emitting Diodes. *Nanoscale* **2022**, *14*, 2771–2779.

(3) Yoo, D.; Bak, E.; Ju, H. M.; Shin, Y. M.; Choi, M. Zinc Carboxylate Surface Passivation for Enhanced Optical Properties of In (Zn) P Colloidal Quantum Dots. *Micromachines* **2022**, *13*, 1775.

(4) Vikram, A.; Zahid, A.; Bhargava, S. S.; Jang, H.; Sutrisno, A.; Khare, A.; Trefonas, P.; Shim, M.; Kenis, P. J. A. Unraveling the Origin of Interfacial Oxidation of InP-Based Quantum Dots: Implications for Bioimaging and Optoelectronics. *ACS Appl. Nano Mater.* **2020**, *3*, 12325–12333.

(5) Organero, J. A.; Tormo, L.; Douhal, A. Caging Ultrafast Proton Transfer and Twisting Motion of 1-Hyroxyl-2-Acetonapthone. *Chem. Phys. Lett.* **2002**, *363*, 409–414.

(6) Douhal, A.; Sanz, M.; Carranza, M. A.; Organero, J. A.; Santos, L. Femtosecond Observation of Intramolecular Charge- and Proton-Transfer Reactions in a Hydroxyflavone Derivative. *Chem. Phys. Lett.* **2004**, *394*, 54–60.

(7) Caballero-Mancebo, E.; Cohen, B.; Moreno, J. M.; Corma, A.; Díaz, U.; Douhal, A. Exploring the Photodynamics of a New 2D-MOF Composite: Nile Red@Al-ITQ-HB. *ACS Omega* **2018**, *3*, 1600–1608.

(8) Gil, M.; Douhal, A. Femtosecond Dynamics of a Non-Steroidal Anti-Inflammatory Drug (Piroxicam) in Solution: The Involvement of Twisting Motion. *Chem. Phys.* **2008**, *350*, 179–185.

(9) Ziółek, M.; Martín, C.; Sun, L.; Douhal, A. Effect of Electrolyte Composition on Electron Injection and Dye Regeneration Dynamics in Complete Organic Dye Sensitized Solar Cells Probed by Time-Resolved Laser Spectroscopy. *J. Phys. Chem. C* **2012**, *116*, 26227–26238.

(10) Omogo, B.; Aldana, J. F.; Heyes, C. D. Radiative and Nonradiative Lifetime Engineering of Quantum Dots in Multiple Solvents by Surface Atom Stoichiometry and Ligands. *J. Phys. Chem. C* **2013**, *117*, 2317–2327.

(11) Jin, S.; Harris, R. D.; Lau, B.; Aruda, K. O.; Amin, V. A.; Weiss, E. A. Enhanced Rate of Radiative Decay in Cdse Quantum Dots upon Adsorption of an Exciton-Delocalizing Ligand. *Nano Lett.* **2014**, *14*, 5323–5328.

(12) Galar, P.; Piatkowski, P.; Ngo, T. T.; Gutiérrez, M.; Mora-Seró, I.; Douhal, A. Perovskite-Quantum Dots Interface: Deciphering Its Ultrafast Charge Carrier Dynamics. *Nano Energy* **2018**, *49*, 471–480.

(13) Eliasson, N.; Rimgard, B. P.; Castner, A.; Tai, C. W.; Ott, S.; Tian, H.; Hammarström, L. Ultrafast Dynamics in Cu-Deficient CuInS2Quantum Dots: Sub-Bandgap Transitions and Self-Assembled Molecular Catalysts. *J. Phys. Chem. C* **2021**, *125*, 14751–14764.

(14) Leatherdale, C. A.; Woo, W. K.; Mikulec, F. V.; Bawendi, M. G. On the Absorption Cross Section of CdSe Nanocrystal Quantum Dots. *J. Phys. Chem. B* **2002**, *106* (31), 7619–7622.
